# Trends and Variations in Colonoscopy Sedation: Insights based on the Dutch Gastrointestinal Endoscopy Audit

**DOI:** 10.1055/a-2942-3921

**Published:** 2026-09-28

**Authors:** Querijn N.E. van Bokhorst, Paula F.B. Vernooij, Karlijn J. Nass, Jos W. Borkent, Jurriën G.P. Reijnders, Peter J. van der Schaar, Marc A.M.T. Verhagen, Paul Fockens, Manon van der Vlugt, Evelien Dekker, on behalf of the Dutch Gastrointestinal Endoscopy Audit scientific committee, Sander van der Beek, Bert A. Bonsing, Sascha C. van Doorn, Marcel J.M. Groenen, Erik J. van Helden, Miangela M. Lacle, Monique E. van Leerdam, Johan E. van Limbergen, Rutger Quispel, Rogier J.J. de Ridder, Manon C.W. Spaander, Roeland Zoutendijk

**Affiliations:** 1 Department of Gastroenterology and HepatologyAmsterdam University Medical Center, Location VU Medical CenterAmsterdamthe Netherlands; 2 Amsterdam Gastroenterology Endocrinology MetabolismAmsterdamthe Netherlands; 3 Cancer Center AmsterdamAmsterdamthe Netherlands; 4 Dutch Institute for Clinical AuditingLeidenthe Netherlands; 5 Department of Gastroenterology and HepatologyRijnstate HospitalArnhemthe Netherlands; 6 Lectorate for Nutrition, Dietetics and Lifestyle, HAN University of Applied SciencesNijmegenthe Netherlands; 7 Department of Gastroenterology and HepatologyErasmus MCRotterdamthe Netherlands; 8 Department of Gastroenterology and HepatologySt. Antonius HospitalNieuwegeinthe Netherlands; 9 Department of Gastroenterology and HepatologyDiakonessenhuisUtrechtthe Netherlands; 10 Department of Gastroenterology and HepatologyWaikato HospitalHamiltonNew Zealand; 11 Department of Internal MedicineRivierenland HospitalTielthe Netherlands; 12 Department of SurgeryLeiden UMCLeidenthe Netherlands; 13 Department of Gastroenterology and HepatologyFlevo HospitalAlmerethe Netherlands; 14 Department of Gastroenterology and HepatologyAdmiraal de Ruyter ZiekenhuisGoesthe Netherlands; 15 Department of PathologyUMC UtrechtUtrechtthe Netherlands; 16 Department of Gastrointestinal OncologyNetherlands Cancer InstituteAmsterdamthe Netherlands; 17 Department of Gastroenterology and HepatologyLeiden UMCLeidenthe Netherlands; 18 Department of Pediatric Gastroenterology and NutritionEmma Children's Hospital, Amsterdam University Medical CentersAmsterdamNetherlands; 19 Department of Gastroenterology and HepatologyReinier de Graaf HospitalDelftthe Netherlands; 20 Department of Gastroenterology and HepatologyMaastricht UMC+Maastrichtthe Netherlands

**Keywords:** endoscopy lower GI tract, quality and logistical aspects, sedation and monitoring

## Abstract

**Background** Sedative medication is routinely used during colonoscopy. Sedation practices may vary in terms of the type and dose of administered medication, possibly impacting clinical outcomes, associated risks, and financial and environmental burdens. We aimed to assess trends and variations in colonoscopy sedation in the Netherlands.

**Methods** Data prospectively recorded in the Dutch Gastrointestinal Endoscopy Audit registry between January 2020 and January 2025 were analyzed. Annual trends in the application of different types of sedation were assessed. Variations across endoscopy units were evaluated by comparing midazolam dosing regimens and the proportional application of propofol-based sedation. Regression analyses assessing the association between sedation and cecal intubation and polyp detection were performed.

**Results** Data from 285,241 colonoscopies were included. Application of propofol-based sedation increased from 5.8% (2020) to 10.0% (2024). The largest increases were observed for IBD-related (11.6–23.2%) and therapeutic indications (28.2–51.7%). For midazolam-based sedation, the mean dose administered varied considerably across units (2.1–5.6mg), as did the proportional application of propofol-based sedation (median 5.0% [IQR 2.3–15.7]; range 0–38%). Compared to midazolam-based sedation using doses equal to or lower than 2.5 mg, propofol-based sedation was not associated with improved cecal intubation (−0.7%; 95% confidence interval [CI] −1.0 to −0.3%), although it was associated with polyp detection (+5.5%; 95% CI +2.8 to +8.3%).

**Conclusion** Colonoscopy sedation practices vary across Dutch endoscopy units in terms of midazolam dosing regimens and the proportional application of propofol-based sedation. In general, propofol-based colonoscopy sedation is increasing. This study’s findings may inform guidelines for more standardized, evidence-based, and resource-conscious sedation practices.

## Introduction

Colonoscopy is the gold standard for diagnosing colorectal diseases. However, patients may perceive colonoscopy as uncomfortable, painful, and embarrassing. Significant procedural discomfort may lead to early procedure termination and the need for repeat examinations. Besides, a negative colonoscopy experience could compromise future participation in screening initiatives, compliance with recommended surveillance schedules, and willingness to undergo diagnostic assessment for colorectal symptoms. 1 2

To optimize patient comfort and procedural tolerability, sedative medication is routinely applied during colonoscopy. Sedation may enhance patient experience, optimize procedural effectiveness, and improve colonoscopy quality in terms of various performance measures. 3 4 Yet, not all studies reported positive associations between sedation and procedural quality. 5 6 7 Besides, sedation also yields various (potential) drawbacks, including prolonged recovery times, an increased risk of adverse events (AEs), higher healthcare costs, and added environmental burden. 8 9 10 11

Ideally, sedation practices are tailored to patient- and procedure-specific factors such as indication, age, comorbidities, and prior colonoscopy experience. Moreover, sedation type and dose should balance patient comfort and procedural efficacy against (potential) drawbacks. In routine practice, however, sedation practices may also be largely shaped by endoscopist preferences, cultural attitudes, available resources, and local policies. This could contribute to substantial variations in standards for colonoscopy sedation, both within and between countries and healthcare systems. 12 13 14 15 16 Such variations may lead to inconsistent patient care and potentially avoidable medication use, costs, and environmental impact.

In the Netherlands, data on sedation practices have been lacking. Accordingly, using data from the Dutch Gastrointestinal Endoscopy Audit (DGEA), this study aimed to: (i) characterize national (annual) trends in colonoscopy sedation, (ii) quantify interunit variation in sedation practices, and (iii) assess associations between sedation type and dose and colonoscopy performance indicators. Obtained insights may inform guidelines for more standardized, evidence-based, and resource-conscious sedation practices.

## Methods

### Study Design and Data Source

This registry-based study used data from the DGEA, a nationwide registry that systematically collects structured patient- and procedure-related data on gastrointestinal endoscopy. This includes data on medication administered during endoscopic procedures.

Data recorded in the DGEA registry are automatically extracted from endoscopy reporting systems at participating endoscopy units, thereby minimizing administrative workload for endoscopists. Automated extraction is enabled through the use of standardized reporting templates, implemented across all participating units in collaboration with the three providers of endoscopy reporting software in the Netherlands. Templates are updated annually following recommendations by the Dutch Society of Gastroenterology. The infrastructure of the DGEA registry has been described in greater detail elsewhere. 17 18 19

In accordance with Dutch law, ethical approval and informed consent were not required for this study as all data were processed anonymously.

### Definition of Study Variables

Colonoscopy sedation was categorized into four groups: “no sedation,” “midazolam and/or opioid-based sedation,” “propofol-based sedation,” and “other”. *Midazolam and/or opioid-based sedation* was defined as the use of midazolam, opioids, or a combination of both. For a subset of study analyses we included all procedures in which midazolam was administered, either with or without opioid coadministration. This subset excluded procedures in which only opioids were administered and is referred to as the “midazolam-based sedation group”. *Propofol* *-based* *sedation* was defined as the use of propofol, with or without use of additional sedative or analgesic agents. The *other* category comprised sedation with agents other than midazolam, opioids, or propofol, such as ketamine.

Trends and variations in sedation practices were analyzed across predefined colonoscopy indication categories: (i) positive fecal immunochemical test (FIT) for the Dutch colorectal cancer (CRC) screening program; (ii) post-polypectomy or CRC surveillance; (iii) familial CRC risk (including hereditary CRC syndromes); (iv) suspected inflammatory bowel disease (IBD) or assessment of IBD disease activity; (v) IBD surveillance; (vi) evaluation of symptoms and other (diagnostic) indications; and (vii) therapeutic procedures.

### Study Outcomes

Primary outcomes encompassed both trends and variations in colonoscopy sedation practices. Trends were assessed through annual changes in the proportion of colonoscopies performed under different types of sedation, with particular focus on propofol-based sedation. Variations were evaluated along two dimensions: first, interunit differences in the mean administered midazolam dose for colonoscopies performed under midazolam-based sedation (with or without opioid coadministration); and second, differences across units in the proportion of procedures performed under propofol-based sedation.

Secondary outcomes included differences in midazolam dosing regimens (mean and distribution) for procedures performed under midazolam-based sedation across colonoscopy indications, as well as differences in cecal intubation and polyp detection rates across procedures performed under different sedation types and using different midazolam doses. Additionally, for procedures performed under midazolam-based sedation (with or without opioid coadministration), exploratory analyses were conducted to assess the correlation between administered midazolam dose (in mg) and the incidence of moderate-to-severe patient discomfort, defined as a Gloucester Comfort Scale (GCS) score of ≥3. 20 As GCS scores were not recorded in the DGEA registry during the study period, this analysis was conducted as a post hoc analysis of a previous study involving 107,328 FIT-positive colonoscopies performed for the Dutch national CRC screening program. 21

### In- and Exclusion Criteria

All colonoscopies recorded in the DGEA registry between January 2020 and January 2025 were screened for eligibility. Procedures performed prior to 2020 were excluded, as older reporting templates did not adequately support registration of data regarding the peri-procedural administration of medication. Procedures lacking a documented indication were excluded as indication was required for stratified analyses. Moreover, follow-up colonoscopies performed within three months of a FIT-positive colonoscopy were excluded given the absence of information regarding the specific reason for these short-term repeat examinations (e.g., residual polyp removal, incomplete procedure).

During the study period, endoscopy report fields pertaining to the peri-procedural administration of medication were not designated as mandatory in the DGEA registry, increasing the risk of inconsistent and incomplete documentation of sedation-related data. To mitigate potential bias arising from inconsistent or incomplete data registry, analyses involving trends and proportional differences in the types of applied sedation were performed including data from endoscopy units that consistently documented administered medication for at least 80% of procedures only. For each unit, data inclusion commenced on the first day of the first month in which this threshold was met (defined as the *valid start date* ; Fig. 1 ) and continued for all subsequent months in which the threshold was maintained through the end of the study period. Within eligible months, individual colonoscopy records without available sedation-related data were excluded.

**Fig. 1:**
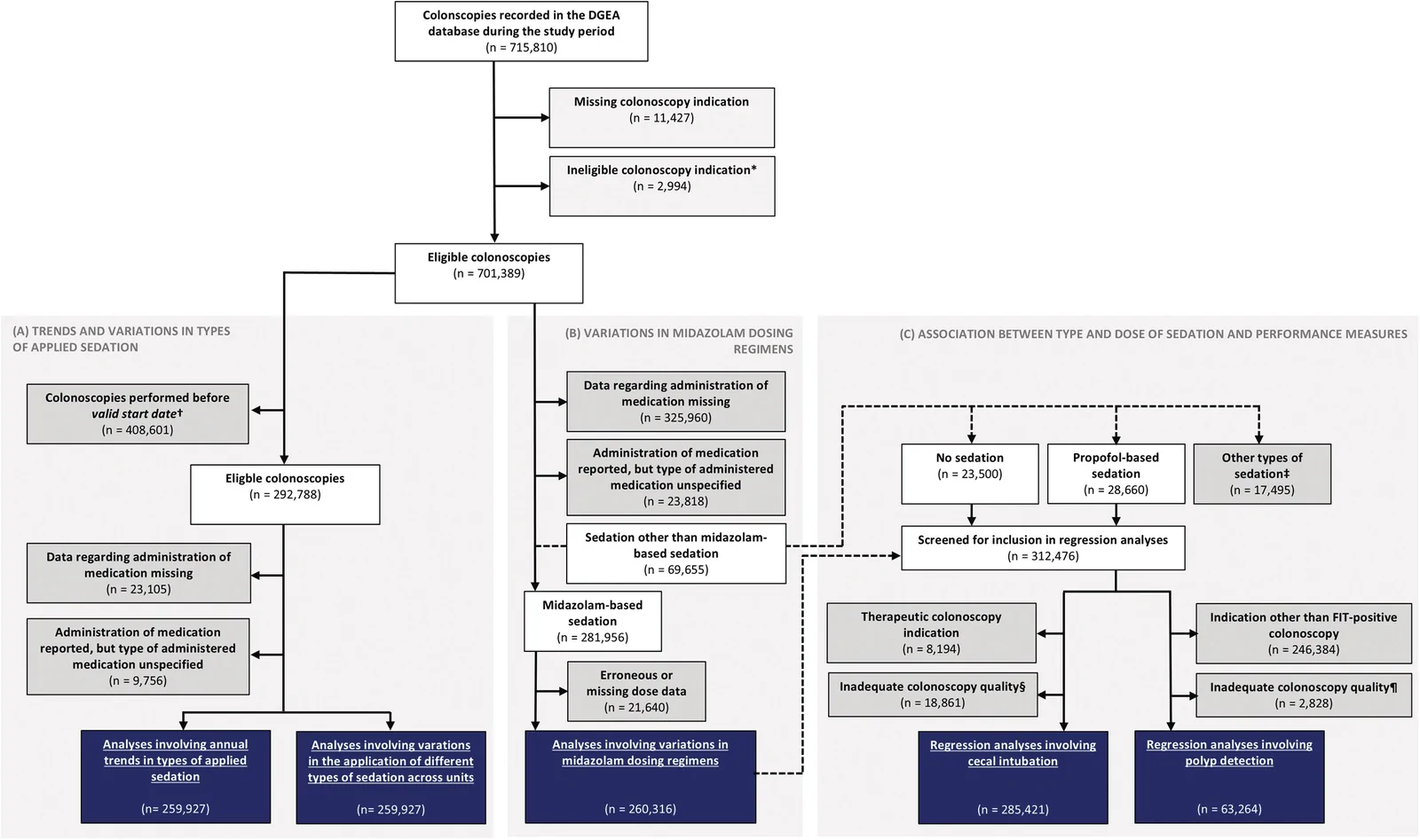
Flowchart illustrating the in- and exclusion of colonoscopies for analyses involving ( **a** ) trends and variations in types of applied sedation, ( **b** ) variations in midazolam dosing regimens, and ( **c** ) the association between type and dose of sedation and performance measures. *Follow-up procedures performed within three months after an initial FIT-positive colonoscopy were excluded due to the absence of information on the specific indications for these short-interval examinations; †For each endoscopy unit, the *valid start date* was defined as the first day of the first month in which information on administered medication was available for at least 80% of procedures; ‡Including sedation using agents other than midazolam or propofol; this includes procedures performed under sedation using opioids only; §Procedures with a Boston Bowel Preparation Scale score <2 in any segment or missing data on the quality of bowel preparation were considered of inadequate quality; ¶Procedures with a Boston Bowel Preparation Scale score <2 in any segment, in which no cecal intubation was established, or missing information regarding these measures were considered of inadequate quality. Abbreviations: DGEA, Dutch Gastrointestinal Endoscopy Audit; FIT, fecal immunochemical test.

For analyses involving variations in dosing regimens, only procedures performed under midazolam-based sedation (with or without opioid coadministration) were included. As the administered midazolam dose (in mg) was required to allow for dose-based stratification, procedures for which the dose of administered midazolam was not recorded were excluded. No dose variation or dose-stratified analyses were performed for propofol-based sedation for two reasons: (i) exact propofol dosing data were not systematically recorded in the DGEA registry; and (ii) propofol dosing is more explicitly weight-based than midazolam dosing, while data regarding patient weight were unavailable.

For analyses involving the association between sedation type or dose and cecal intubation and polyp detection rates, only procedures performed under midazolam-based sedation and propofol-based sedation were included. Procedures performed under sedation using opioids only were excluded from these analyses, as well as from the analyses involving variations in dosing regimens, as—owing to problems with the configuration of reporting templates—data on opioid administration and dosing were inconsistently reported for procedures performed prior to January 2024.

For analyses involving cecal intubation, procedures with a therapeutic indication, inadequate bowel preparation (defined as a Boston Bowel Preparation Scale score <2 in any segment), or missing bowel preparation data were excluded. Analyses involving polyp detection were restricted to FIT-positive colonoscopies, as this was the only indication category for which the recording of polyp detection was mandatory throughout the complete study period. Additionally, procedures without cecal intubation, those with inadequate bowel preparation, or those with missing data on either measure were excluded from these analyses.

Finally, to reduce the risk of biased estimates due to small sample sizes, endoscopy units were included in analyses only if they recorded at least 50 (trends and variations in the types of applied sedation) or 100 (variations in midazolam dosing regimens) procedures within a given indication category in a specific year.

### Statistical Analyses

Patient and procedure characteristics were summarized using descriptive statistics. Normality was assessed using histograms and QQ-plots. Annual trends in the application of different types of sedation were evaluated using linear regression, with sedation type as a binary outcome and year as a continuous predictor. Although logistic regression is typically used for dichotomous outcomes, it provides ratios (relative measures) rather than absolute percentual changes. Linear regression was therefore employed. As normality assumptions were violated in the linear regression analyses, 95% confidence intervals (CIs) were derived via bootstrapping with a 1000 iterations. 22

We also assessed whether type of sedation (independent variable) was related to successful cecal intubation and polyp detection (dependent dichotomized variables). Although logistic regressions providing odds ratios (ORs) are mostly used for these types of analyses, ORs may overestimate the magnitude of the association between exposure and outcome in high-incidence settings (e.g., polyp detection). Therefore, we used Poisson regression with a log link to estimate incidence rate ratios (IRRs), which are less prone to bias in these settings. 22 To obtain robust standard errors and account for clustering of observations within endoscopy units, we applied generalized estimating equations with an exchangeable correlation structure. IRRs were interpreted as percentual differences in incidence rates (e.g., an IRR of 1.06 indicates a 6% higher incidence compared with the reference category).

For regression analyses, midazolam-based sedation was categorized into three groups based on the dose administered: a dose equal to or lower than 2.5 mg ( < 2.5 mg), a dose equal to or between 2.6 and 5 mg (2.6–5 mg), and a dose higher than 5 mg (>5 mg). Following the results of a previous DGEA registry study, analyses involving cecal intubation were performed with adjustment for patient age, gender, American Society of Anesthesiologists (ASA) score, and colonoscopy indication (FIT-positive vs. surveillance [post-polypectomy, post-CRC, IBD and familial CRC risk surveillance] vs. symptoms and other [including all non-surveillance IBD indications]). Analyses involving polyp detection were performed with adjustment for patient age, gender, and ASA score. 23

To assess the association between midazolam dosing regimens and patient comfort, we performed a Pearson correlation analysis correlating the mean midazolam dose administered (calculated on the level of individual endoscopy units) with the proportion of patients reporting moderate-to-severe discomfort (GCS > 3). 20 Correlation strength was interpreted following the recommendations by Schober et al. 24

All analyses were performed using R version 4.4.1 (R Foundation for Statistical Computing, Vienna, Austria).

## Results

A total of 715,810 colonoscopies were recorded in the DGEA registry during the study period. After applying eligibility criteria, 259,927 colonoscopies remained for analyses involving trends and variations in the types of sedation applied. A total of 260,316 procedures were included in analyses involving variations in midazolam dosing regimens for procedures performed under midazolam-based sedation ( Fig. 1 ). The number of endoscopy units contributing eligible data for analyses involving both trends and variations increased annually (minimum of 10 in 2020 and maximum of 48 in 2024; **Fig. S1** ). Characteristics of included colonoscopies are reported in **Table S1** .

### Trends in Types of Applied Sedation

Overall trends in types of applied colonoscopy sedation are depicted in Fig. 2 . The proportion of procedures performed without sedation remained relatively stable throughout the study period (range: 1.1–2.1%; annual trend: −0.2% [95% CI −0.3% to −0.2%]). The application of midazolam- and/or opioid-based sedation gradually declined from 93% in 2020 to 89% in 2024 (annual trend: -1.0% [95% CI −1.1% to −0.9%]), while the application of propofol-based sedation increased from 5.8% to 10.0% (annual trend: +1.1% [95% CI +1.0% to +1.2%]). This upward trend in propofol-based sedation was observed across all indication categories, with the largest increases seen for IBD-related (11.6%–23.2%; annual change: +2.7% [95% CI +2.0% to +3.3%]) and therapeutic indications (28.2%–51.7%; annual change: +5.3% [95% CI +4.3% to +6.3%]). Indication-specific trends in the application of propofol-based sedation are illustrated in Fig. 3 and further detailed in **Table S2** .

**Fig. 2:**
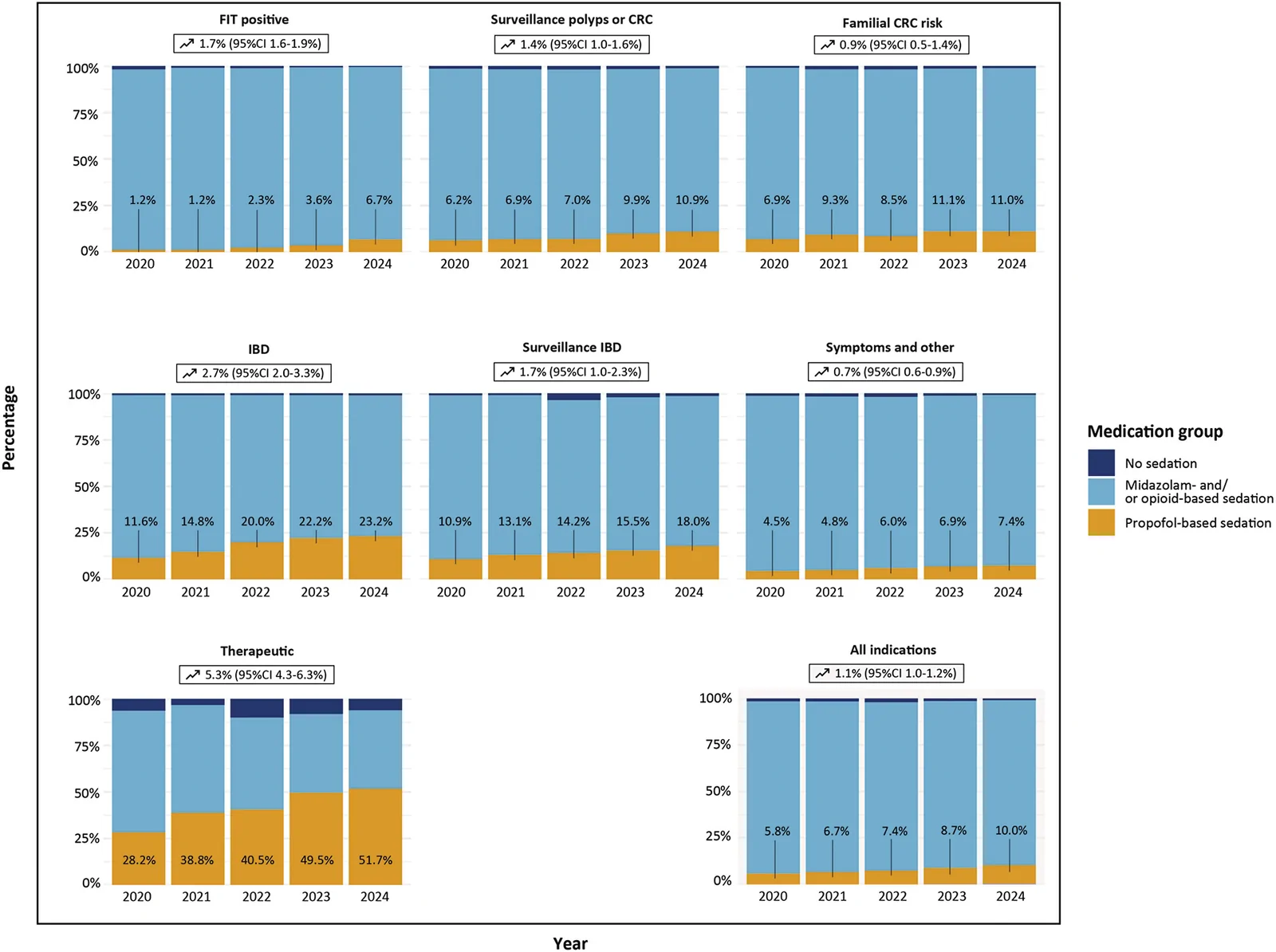
Trends in colonoscopy sedation practices between 2020 and 2024, stratified by indication category. Percentages with corresponding 95% confidence intervals shown above each panel represent the estimated annual change in the proportion of procedures performed under propofol-based sedation. The percentages within each column denote the proportion of procedures performed under propofol-based sedation. Note: owing to its small sample size, the “other” group was excluded from this figure. Abbreviations: FIT, fecal immunochemical test; CI, confidence interval; CRC, colorectal cancer; IBD, inflammatory bowel disease.

**Fig. 3:**
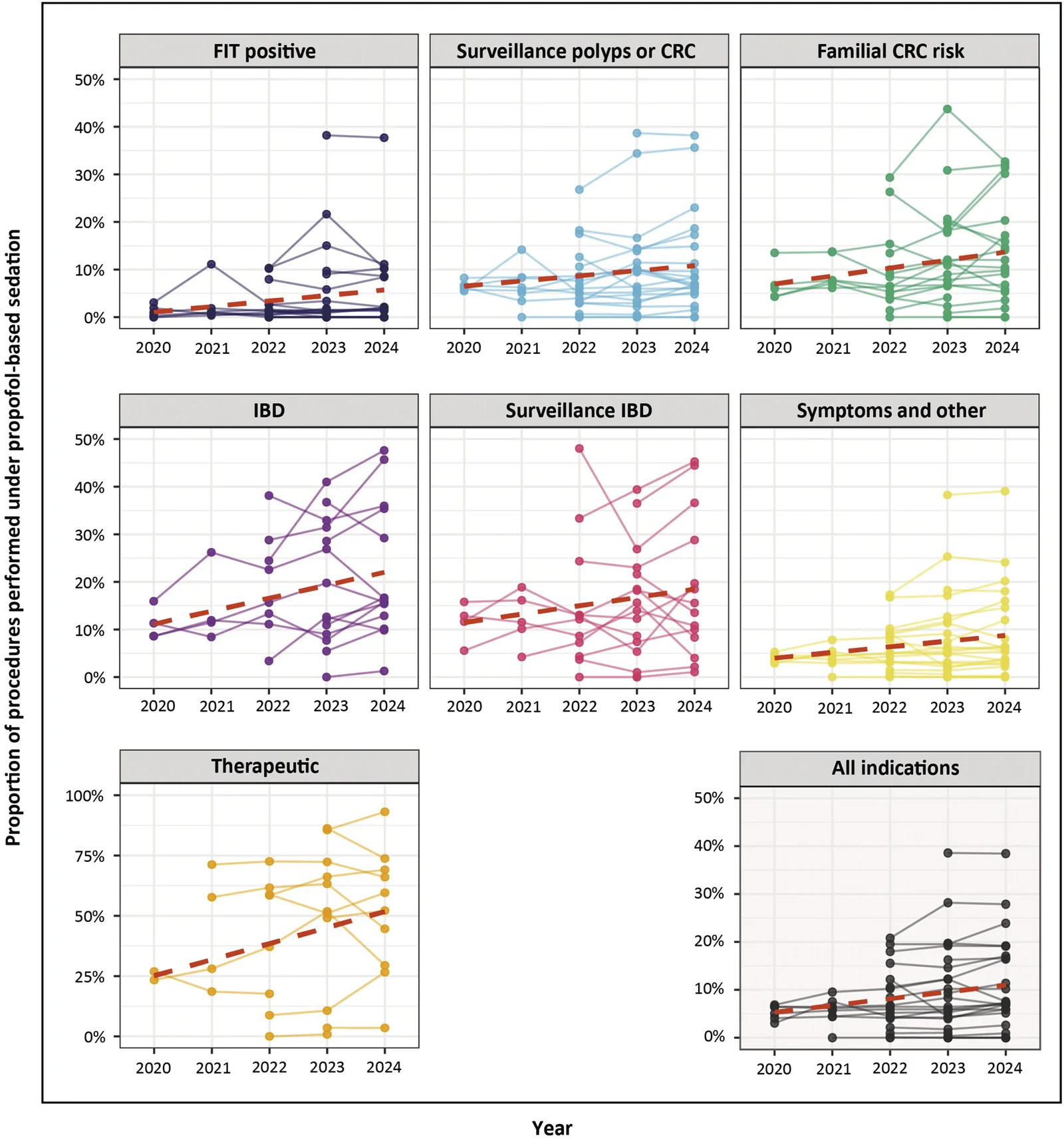
Unit-specific trends (2020–2024) and interunit variation in the proportion of procedures performed under propofol-based sedation, stratified by indication category. Each line represents an individual endoscopy unit. Data are shown for units with at least 50 procedures recorded within a given indication category per calendar year and with available data for at least two consecutive years only. Red dashed lines denote the overall change in the proportion of procedures performed under propofol-based sedation between 2020 and 2024. Abbreviations: FIT, fecal immunochemical test; CRC, colorectal cancer; IBD, inflammatory bowel disease.

### Variations in the Application of Propofol-based Sedation

The median proportion of procedures performed under propofol-based sedation was 5.0% (IQR 2.3–15.7%) across units, ranging from 0 to 38% ( **Table S3** ). Interunit variations are illustrated in Fig. 3 . As propofol-based sedation may be associated with colonoscopy indication, the proportional distribution of performed procedures across indication categories per unit is illustrated in **Fig. S2** . IBD-related and IBD surveillance indications, the indications for which the most pronounced interunit variation in the use of propofol-based sedation was observed ( Fig. 3 ; **Table S3** ), accounted for less than 10% of procedures in the vast majority of units (34/40 units, 85%; **Fig. S2** ).

### Variations in Midazolam Dosing Regimens

For procedures performed under midazolam-based sedation (with or without opioid coadministration), mean and median midazolam doses administered across colonoscopies with different indications are illustrated in Fig. 4 . Overall, administered doses were lowest for post-polypectomy or CRC surveillance indications (3.4 ± 1.3 mg) and highest for IBD-related indications (4.3 ± 1.6 mg).

**Fig. 4:**
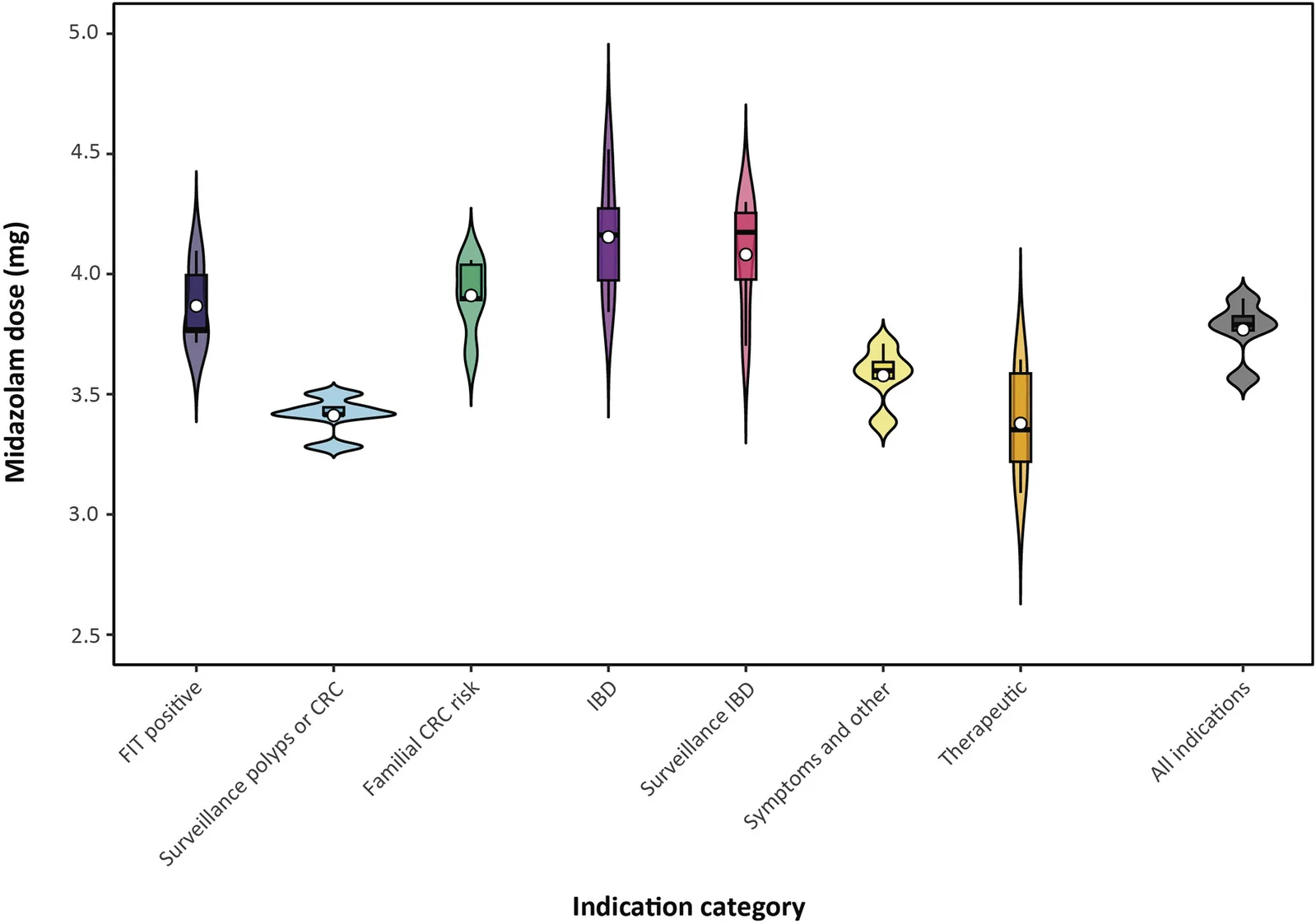
Violin plots displaying midazolam doses as applied for midazolam-based sedation (either with or without coadministration of opioids) across different colonoscopy indication categories. Each violin illustrates the kernel density distribution of the administered doses, with an embedded boxplot showing the median and interquartile range. The white dots denote the mean dose for each category. The width of the violins reflects the distributional spread, allowing visual comparison of variation across categories. Abbreviations: FIT, fecal immunochemical test; CRC, colorectal cancer; IBD, inflammatory bowel disease.

Interunit variations in the mean midazolam dose administered are illustrated in Fig. 5 , with unit-level means ranging from 2.1 ± 1.2 to 5.6 ± 1.7 mg. Mean doses stratified by indication category and endoscopy unit are presented in **Table S4** . Annual trends across indication categories are depicted in **Fig. S3** .

**Fig. 5:**
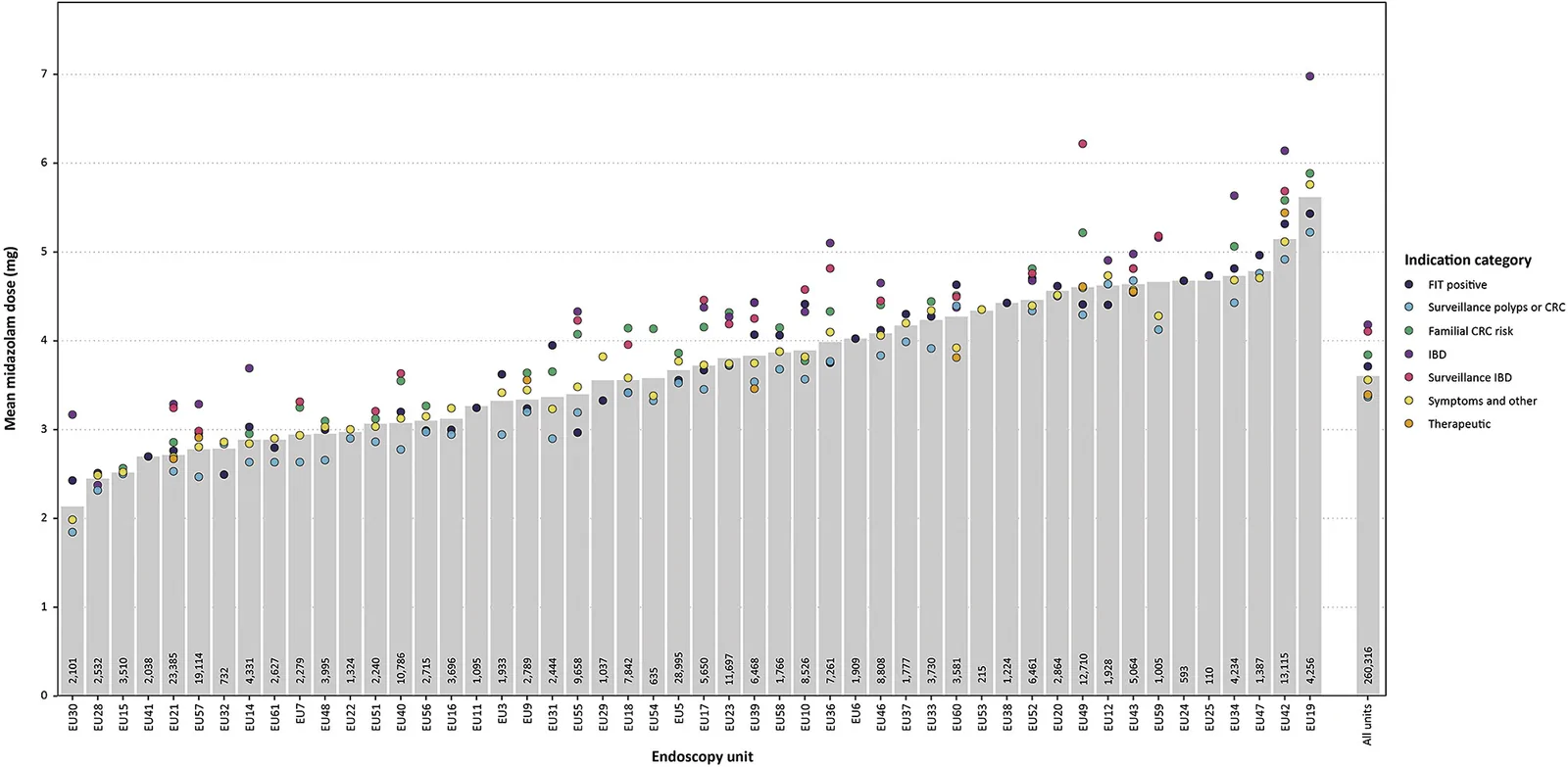
Interunit variation in the mean dose of midazolam administered for procedures performed under midazolam-based sedation (either with or without opioid coadministration). Each gray bar represents an individual endoscopy unit, with bar height indicating the overall mean midazolam dose administered during colonoscopies performed at that unit. Endoscopy units are ordered from left (lowest) to right (highest) based on their overall mean midazolam dose administered. Colored dots superimposed on the bars depict indication-specific mean midazolam doses. Numbers at the base of each bar indicate the total number of colonoscopies included per endoscopy unit. Indication-specific means were calculated and displayed only when at least 100 colonoscopies were available for a given indication within an endoscopy unit; categories with fewer than 100 procedures were excluded. Abbreviations: FIT, fecal immunochemical test; CRC, colorectal cancer; IBD, inflammatory bowel disease.

### Association between Sedation and Cecal Intubation and Polyp Detection

A total of 285,421 and 63,264 colonoscopies were included in regression analyses involving cecal intubation and polyp detection, respectively ( Fig. 1 ). Results are reported in Table 1 . Using midazolam-based sedation with doses < 2.5 mg as the reference group, no sedation was associated with a lower incidence of both successful cecal intubation (−3.5% [95% CI −3.9% to −3.1%]) and polyp detection (−5.9% [95% CI −8.1% to −4.6%]). A midazolam dose between 2.6 and 5 mg was associated with a small increase in cecal intubation (+0.6% [95% CI +0.4% to +0.7%]) and polyp detection (+2.1% [95% CI +0.9% to +3.3%]). Midazolam doses over 5 mg and propofol-based sedation did not improve cecal intubation rates but were associated with higher polyp detection rates (up to +5.5% [95% CI +2.8% to +8.3%]; Table 1 ).

**Table 1:** Multivariable regression analyses evaluating the effect of sedation type and dose on cecal intubation and polyp detection.

Cecal intubation				
	N	CIR (%), unadjusted	IRR (95% CI) of successful cecal intubation, adjusted*	Percentual difference in incidence rate (95% CI)†
**Sedation**				
None	19,176	92.4	0.965 (0.961–0.969)	−3.5 (−3.9 to −3.1)
Midazolam-based sedation (dose < 2.5 mg)‡	85,062	95.1	Reference	Reference
Midazolam-based sedation (dose 2.6–5 mg)‡	151,091	97.5	1.006 (1.004–1.007)	+0.6 (+0.4–+0.7)
Midazolam-based sedation (dose >5 mg)‡	7,550	95.9	0.997 (0.992-1.002)	–0.3 (–0.8 to +0.2)
Propofol-based sedation	22,542	94.3	0.993 (0.990–0.997)	–0.7 (–1.0 to –0.3)
**Gender**				
Male	139,639	96.2	Reference	Reference
Female	145,782	95.1	0.989 (0.987–0.991)	–1.1 (–1.3 to –0.9)
**Age**				
< 55	59,701	96.0	1.013 (1.010–1.015)	+1.3 (+1.0 to +1.5)
56–65	89,545	96.9	1.008 (1.006–1.010)	+0.8 (+0.6–+1.0)
66–75	92,966	95.6	Reference	Reference
>75	43,209	92.6	0.983 (0.980–0.986)	–1.7 (–2.0 to –1.4)
**ASA score**				
I	199,808	95.6	Reference	Reference
II	59,999	97.3	0.989 (0.988–0.991)	-1.1 (-1.2 to –0.9)
> III	25,614	92.3	0.966 (0.962–0.970)	−3.4 (−3.8 to −3.0)
**Colonoscopy indication**				
FIT positive (CRCSP)	64,362	98.3	Reference	Reference
Surveillance§	91,613	96.1	0.983 (0.981–0.985)	–1.7 (–1.9 to –1.5)
Symptoms and other	129,446	93.9	0.960 (0.958–0.961)	–4.0 (–4.2 to −3.9)
**Polyp detection**				

	N	PDR (%), unadjusted	IRR (95% CI) of polyp detection, adjusted¶	Percentual difference in incidence rate (95% CI)†
**Sedation**				
None	3,502	69.4	0.941 (0.919–0.964)	−5.9 (–8.1 to –4.6)
Midazolam-based sedation (dose < 2.5 mg)‡	14,667	72.4	Reference	Reference
Midazolam-based sedation (dose 2.6–5 mg)‡	35,925	73.4	1.021 (1.009–1.033)	+2.1 (+0.9–+3.3)
Midazolam-based sedation (dose >5 mg)‡	1,205	72.4	1.031 (0.995–1.069)	+3.1 (–0.5 to +6.9)
Propofol-based sedation	2,449	76.0	1.055 (1.028–1.083)	+5.5 (+2.8–+8.3)
**Gender**				
Male	31,825	77.2	Reference	Reference
Female	25,923	67.8	0.873 (0.864–0.882)	–12.7 (–13.6 to –11.8)
**Age**				
< 65	31,235	70.0	Reference	Reference
>65	26,513	76.2	1.073 (1.062–1.084)	+7.3 (+6.2–+8.4)
**ASA score**				
I	14,234	68.4	Reference	Reference
II	39,677	74.5	1.069 (1.055–1.083)	+6.9 (+5.5–+8.3)
> III	3,837	74.8	1.054 (1.029–1.079)	+5.4 (+2.9–+7.9)

CIR, cecal intubation rate; IRR, incidence rate ratio; ASA, American Association of Anesthesiologists; FIT, fecal immunochemical test; CRCSP, colorectal cancer screening program; PDR, polyp detection rate. *Adjusted for patient gender, age, ASA score, and colonoscopy indication; †Percentual difference in incidence rate is calculated from the IRR: (IRR − 1) × 100%; ‡Either with or without coadministration of opioids; §Including all colonoscopies for post-polypectomy, post-CRC or IBD surveillance, as well as (surveillance) procedures in patients with familial CRC risk; ¶Adjusted for patient gender, age, and ASA score. Note: analyses were performed with exclusion of procedures for which any of the variables included in the regression model were missing.

### Association between Midazolam Dosing Regimens and Patient Comfort

After excluding procedures with missing data, procedures during which no midazolam was administered, and procedures from units with fewer than 100 cases, data from 72,768 procedures across 87 endoscopy units remained for analysis. A weak correlation was observed between the mean midazolam dose administered and the incidence of moderate-to-severe patient discomfort (Pearson’s *r* = −0.11 [95% CI, −0.31 to 0.11]) ( **Fig. S4** ). 24

## Discussion

This study is the first to systematically examine national trends and interunit variations in colonoscopy sedation practices in the Netherlands. It demonstrates a marked increase in the proportion of colonoscopies performed under propofol-based sedation, reaching up to 10% nationwide at this point. Moreover, substantial heterogeneity was observed across endoscopy units, both in terms of the proportion of procedures performed under propofol-based sedation (0–38%) and in terms of midazolam dosing regimens for procedures performed under midazolam-based sedation (mean doses ranging from 2.1 to 5.6 mg).

Findings of the current study align with the global trend of increased application of propofol-based sedation in gastrointestinal endoscopy. 25 This shift may be driven by greater availability and reduced costs of propofol. In addition, the expanding implementation of nurse-administered propofol sedation (NAPS), demonstrated to be a safe and effective alternative to anesthesia-directed sedation 26 27 , reduces reliance on specialized sedation practitioners and broadens opportunities for propofol-based sedation. Furthermore, clinicians may more frequently opt for propofol-based sedation considering that it may result in higher patient satisfaction compared to midazolam- and/or opioid-based sedation. 27 Finally, it should be considered that, with broader availability of sedative and analgesic treatment options, patient tolerance for procedural discomfort may have decreased over recent years.

The increase of propofol-based sedation may prove particularly beneficial for patients with chronic conditions such as IBD, who require relatively frequent endoscopic examinations and often experience greater procedural burden. 28 29 This may, in part, also explain the pronounced annual increase in propofol-based sedation observed for IBD-related colonoscopies in the current study (+2.7% annually; Figure 2 ). The largest annual increase was, however, observed for therapeutic procedures (+5.3% annually; Figure 2 ). The latter likely reflects the growing adoption of interventional endoscopic procedures as an alternative to surgery, typically requiring propofol-based sedation because of their longer duration and increased technical complexity. 30

In terms of variations in sedation practices, previous studies highlighted considerable differences both between countries and healthcare systems 12 13 , as well as across endoscopy units within them. 14 15 16 Differences between healthcare systems may primarily reflect divergent guideline recommendations 12 , whereas differences within healthcare systems may be more attributable to factors such as patient case-mix (e.g., academic centers vs. outpatient clinics). However, for indications such as FIT-positive and post-polypectomy surveillance colonoscopies, patient populations can be considered to be very comparable across units. Accordingly, the observed interunit variations for these indications ( Figs. 3 and 5 ) suggest that sedation regimens may often be more strongly influenced by endoscopists’ preferences than by patient-related or procedural factors. On the other hand, these differences may, in part, also be driven by available resources.

In appraising optimal approaches for colonoscopy sedation, several factors merit consideration. Primarily, although midazolam-based sedation (either with or without opioid coadministration) is employed for the vast majority of colonoscopies in the Netherlands, insights into the association between midazolam dosing regimens and patient comfort remain limited. The present study demonstrated a weak correlation between midazolam dose and patient comfort ( **Fig. S4** ), corroborating the findings of the single study previously evaluating this association. 15 These findings suggest that factors such as endoscopists’ technical proficiency and patient-specific characteristics may exert a greater influence on patient comfort than midazolam dosing regimens.

Second, sedation may influence colonoscopy quality. In the present study, sedation was associated with higher cecal intubation rates compared to unsedated procedures ( Table 1 ). Moreover, midazolam doses above 2.5 mg were associated with a marginal increase in cecal intubation rates compared to doses below 2.5 mg (+0.6% [95% CI +0.4% to +0.7%]), while propofol-based sedation conferred no additional improvement over low-dose (i.e., < 2.5 mg) midazolam-based sedation ( Table 1 ). The latter is consistent with a recent review of 33 studies demonstrating no clear advantage of propofol-based sedation over midazolam and/or opioid-based sedation in terms of cecal intubation. 27 These results should, however, be interpreted with some caution as more technically challenging procedures may necessitate deeper levels of sedation.

Polyp detection rates in FIT-positive colonoscopies were comparable between unsedated procedures and those performed under low-dose (i.e., < 2.5 mg) midazolam-based sedation in our study. In contrast, midazolam doses over 2.5 mg and propofol-based sedation were associated with increased polyp detection ( Table 1 ). Yet, the clinical implications of this finding should be carefully interpreted given the modest absolute gain (up to +5.5%; Table 1 ), the limitations inherent to the polyp detection rate as a colonoscopy quality indicator 31 , and the lack of supporting evidence from prior studies assessing the effect of propofol-based sedation on both the polyp detection rate and the, more clinically relevant, adenoma detection rate. 11 32 33

Third, different sedation regimens are associated with divergent financial and environmental burdens. Recent analyses from Canada and the United States estimated that propofol-based sedation adds approximately $127 CAD and $182–233 USD per case compared to midazolam- and/or opioid-based sedation 10 11 , primarily due to the need for specialized sedation practitioners. Going forward, the gradual implementation of NAPS may help mitigate some of these costs. 26 27 Additionally, while sedation contributes only a minor share to the overall carbon dioxide (CO₂) emissions from colonoscopy, 34 1 g of midazolam and propofol are associated with emissions of approximately 1–2 and 4–6 g CO₂ per procedure, respectively. 35 While individually modest, these differences may carry some relevance given the high annual volumes of endoscopic procedures performed worldwide. Also, it should be noted that these figures reflect only cradle-to-gate emissions associated with medication production and exclude emissions related to the materials used for their administration.

In light of the considerations above, endoscopy units may benefit from critically appraising their (standard) sedation practices. Regarding midazolam-based sedation, the routine administration of relatively high starting doses (i.e., midazolam doses exceeding 2.5 mg) warrants careful consideration: beyond the potentially limited benefit for patient comfort and procedural quality ( Fig. 5 , Table 1 ), higher midazolam doses may be associated with greater financial and environmental burden and increase the risk of sedation-related AEs. 8 9 10 11 Adoption of lower standard starting doses (e.g., 2.5 mg or lower) with cautious titration based on patient- and procedure-specific characteristics and the occurrence of significant peri-procedural discomfort should therefore be encouraged.

With respect to propofol-based sedation, more frequent application in patients with chronic conditions, relevant comorbidities, experiencing high procedural burden, or requiring therapeutic interventions, may be considered a logical and beneficial development. Conversely, routine application in patients without relevant comorbidities or risk factors for significant peri-procedural discomfort, particularly those undergoing a single screening procedure for indications such as symptom evaluation, warrants careful consideration; increased availability alone may not justify routine application of propofol-based sedation. This is supported by the limited and somewhat ambiguous evidence regarding the benefits of propofol-based sedation on procedural quality compared to midazolam- and/or opioid-based sedation ( Table 1 ) 11 27 32 33 , its associated increase in risk of AEs, 9 and its relatively higher financial and environmental burden. 10 11 34 36

This study has several strengths. First, it draws on one of the largest real-world colonoscopy cohorts in which trends and variations in sedation practices have been evaluated, providing a useful benchmark for other countries and healthcare systems. Second, the multivariable analyses examining sedation regimens in relation to colonoscopy performance metrics may offer valuable insights for clinical practice.

The principal limitation of this study involves the inconsistent availability of data involving the (co-)administration of opioids across records dating from before January 2024. This resulted in a potential underestimation of the proportion of procedures performed under sedation with opioids only, as well as the inability to account for opioid coadministration alongside midazolam and propofol in various study analyses. To further characterize this limitation, we performed exploratory analyses of data collected after optimization of the data infrastructure (starting from January 2024; *n* = 95,188 colonoscopies), which revealed that opioid-only sedation was applied in 8.4% of procedures and that midazolam-based sedation was combined with opioids in the vast majority (93%) of cases. As sedation practices in 2024 are likely representative of those in preceding years, the high rate of opioid coadministration (93%) suggests that the results pertaining to the midazolam-based sedation group in the current study are largely generalizable to combined midazolam-opioid sedation regimens. Furthermore, while midazolam doses were slightly lower when opioids were coadministered compared to midazolam alone, differences remained at or below 0.5 mg across most indication categories ( **Fig. S5** ). Similar analyses involving coadministration of analgesics alongside propofol were not performed at this point, particularly as consistent propofol dosing data were unavailable.

Another limitation relates to the fact that during the early years of the study period only a subset of endoscopy units contributed eligible data, which may have introduced some degree of inclusion bias. Moreover, we did not perform analyses involving the association between sedation and occurrence of AEs. This relates to the fact that the Dutch Registration of Complications in Endoscopy 18 lacks specific information on whether an AE was primarily attributable to procedural sedation, as well as the fact that transient sedation-related events (e.g., oxygen desaturation) are likely underreported. Furthermore, the DGEA did not include measures of patient-reported comfort during the study period, which we attempted to address through an exploratory analysis using an alternative dataset. 15 Additionally, data on procedure duration was not recorded in the DGEA registry during the study period. As longer procedures may require administration of additional sedatives, this represents a potential source of bias. However, the impact is likely limited given the large number of included procedures, which reduces the influence of any subset of procedures with atypically long durations. The DGEA registry does also not include information on the experience of the endoscopist performing each procedure, which precluded adjustment for endoscopist experience as a potential confounder in the analyses involving cecal intubation and polyp detection. Finally, in light of findings from earlier studies 21 , the reported proportion of procedures during which no sedation was applied likely represents an underestimation, which may be attributable to incomplete registration of sedation-related data (use of sedation: yes vs. no) whenever no sedation was applied.

As a final remark, the effect of a given dose of a sedative agent may vary considerably between patients depending on factors such as age, weight, concomitant medication use, and individual pharmacogenomic variation, potentially resulting in meaningful interindividual differences in sedation depth for equivalent doses. Meanwhile, sedation depth, quantifiable through measures such as the Leeds Sedation Scale 20 , was not recorded in the DGEA database during the study period and therefore could not be accounted for in our analyses. Nevertheless, the potential impact of such interindividual variation may be considered limited, given that the large patient cohort attenuates the influence of individual differences.

Considering the limitations outlined above, the infrastructure for recording medication-related data in the DGEA registry was improved in 2024, particularly allowing for more accurate and consistent data collection involving administration of opioids. Furthermore, as of January 2025, the GCS score was introduced as a mandatory colonoscopy quality indicator in the DGEA registry. Meanwhile, as the diagnostic accuracy of the GCS in reflecting patient experience can be debated 20 , future implementation of variables representing patient-reported experience is considered. Additionally, implementation of procedure duration, Leeds Sedation Scale score 20 , and a dedicated variable to capture peri-procedural sedation-related AEs (e.g., desaturation, requirement for pharmacological antagonists, and premature procedure termination) will be considered for future DGEA registry updates.

In conclusion, considerable variation exists in colonoscopy sedation practices across Dutch endoscopy units. Given the limited incremental benefit of higher midazolam doses on procedural quality, lower standard starting doses with careful, individualized titration based on patient- and procedure-specific factors may be preferable for midazolam-based sedation. Besides, the proportion of procedures performed under propofol-based sedation is increasing, which may be advantageous for patients undergoing more burdensome procedures or requiring therapeutic interventions. However, the routine application of propofol-based sedation for diagnostic colonoscopy indications should be carefully balanced against its relatively higher financial, logistical, and environmental burden. The findings of this study may inform future guidelines for more standardized, evidence-based, and resource-conscious sedation practices.

## Declaration of GenAI Use

During the preparation of this work, the authors used Claude (Anthropic) for language editing. All content was reviewed and approved by the authors, who take full responsibility for the final manuscript.

## Supplementary Material

Ergänzendes Material Supplementary Material
