# Supplementary material for: Trends and Variations in Colonoscopy Sedation: Insights based on the Dutch Gastrointestinal Endoscopy Audit
Source: Endosc Int Open. 2026 Sep 28;14:a29423921. doi: 10.1055/a-2942-3921 (PMC13643738; doi:10.1055/a-2942-3921)
Supplement: Supplementary file 1 [file 10-1055-a-2942-3921_29675403.pdf]

SUPPLEMENTARY MATERIALS

Trends and variations in colonoscopy sedation: insights based on the Dutch Gastrointestinal Endoscopy Audit

Querijn N.E. van Bokhorst[1,2,3,4], Paula F.B. Vernooij[1,2,3,4], Karlijn J. Nass[5], Jos W. Borkent[6], Jurriën G.P. Reijnders[7], Peter J. van der Schaar[8], Marc A.M.T. Verhagen[9], Paul Fockens[1,2,3,10], Manon van der Vlugt[1,2,3], Evelien Dekker[1,2,3] on behalf of the Dutch Gastrointestinal Endoscopy Audit scientific committee

Table S1. Patient and colonoscopy characteristics

	Analyses involving trends and variations in types of applied sedation n (%)	Analyses involving variations in midazolam dosing regimens, n (%)
**All colonoscopies**	259,927	260,316
**Patient characteristics**		
Gender		
Male	125,863 (48)	127,025 (49)
Female	134,027 (52)	133,261 (51)
Missing or unknown	37 (<1)	30 (<1)
Patient age in years, median (IQR)	65 (56-73)	65 (56-73)
ASA classification		
I – Normal healthy patient	55,934 (22)	55,505 (21)
II – Mild systemic disease	177,773 (68)	184,436 (71)
III – Severe systemic disease that is not a constant threat to life	24,421 (9.4)	18,946 (7.3)
IV – Severe systemic disease that is a constant threat to life	363 (<1)	137 (<1)
V – Moribund patient not expected to survive >24 hours with or without intervention	6 (<1)	4 (<1)
Missing	1,430 (<1)	1,288 (<1)
**Procedure characteristics**		
Indication		
FIT positive		
Primary colonoscopy after a positive FIT for the CRCSP	47,502 (18)	58,004 (22)
Surveillance polyps or CRC		
Surveillance – adenoma	45,067 (17)	40,882 (16)
Surveillance – CRC	14,441 (5.5)	15,206 (5.8)
Surveillance – after excision polyp	1,737 (<1)	1,620 (<1)
Familial risk		
Familial risk – polyposis coli	868 (<1)	808 (<1)
Familial risk – CRC	7,975 (3.1)	7,377 (2.8)
Familial risk – HNPCC/Lynch syndrome	2,970 (1.1)	3,194 (1.2)
Familial risk - undefined	1,550 (<1)	1,530 (<1)
IBD		
Suspected IBD	4,910 (1.9)	3,907 (1.5)
Evaluation of disease activity IBD	4,235 (1.6)	3,366 (1.3)
IBD surveillance		
Surveillance – IBD	8,223 (3.2)	8,033 (3.1)
Symptoms and other		
Changed stools	29,138 (11)	27,738 (11)
Rectal blood loss	31,503 (12)	30,975 (12)
Iron deficiency anemia	17,804 (6.8)	17,606 (6.8)
Chronic diarrhea	7,022 (2.7)	7,546 (2.9)
Abdominal pain	23,060 (8.9)	22,914 (8.8)
Incidental finding at radiological or perianal examination	5,407 (2.1)	5,859 (2.3)

Endosc Int Open | DOI 10.1055/a-2942-3921 | © The Author(s).

Positive FIT through general practitioner	932 (<1)	775 (<1)
Therapeutic	5,583 (2.1)	2,976 (1.1)
Quality of bowel preparation*		
Adequate	249,143 (96)	248,831 (96)
Inadequate	10,641 (4.1)	11,485 (4.4)
Missing	143 (<1)	0
Sedation		
No sedation	3,764 (1.4)	0
Midazolam and/or opioid-based sedation	233,480 (90)	260,316 (100)
Propofol-based sedation	22,280 (8.6)	0
Other	403 (<1)	0

IQR, interquartile range; ASA, American Association of Anesthesiologists; FIT, fecal immunochemical test; CRCSP, colorectal cancer screening program; CRC, colorectal cancer; HNPCC, hereditary nonpolyposis colorectal cancer; IBD, inflammatory bowel disease. *Adequate bowel preparation was defined as a Boston Bowel Preparation Scale score $\geq$2 in all relevant segments (excluding segments that could not be scored due to factors such as previous surgery).

Table S2. Trends in sedation practices for different categorized colonoscopy indications

	2020	2021	2022	2023	2024
**FIT positive**					
**Total colonoscopies, n**	**2,202**	**3,260**	**7,689**	**17,337**	**17,014**
**Unique endoscopy units, n**	**7**	**9**	**17**	**26**	**30**
No medication, n (%)	40 (1.8)	34 (1.0)	95 (1.2)	139 (0.8)	91 (0.5)
Mild sedation, n (%)	2,136 (97)	3,187 (98)	7,418 (97)	16,580 (96)	15,768 (93)
Deep sedation, n (%)	6 (1.2)	39 (1.2)	176 (2.3)	617 (3.6)	1,152 (6.8)
Other, n (%)	0	0	0	1 (<1)	3 (<1)
**Surveillance polyps or CRC**					
**Total colonoscopies, n**	**3,461**	**5,612**	**11,484**	**18,258**	**22,430**
**Unique endoscopy units, n**	**7**	**8**	**21**	**28**	**36**
No medication, n (%)	52 (1.5)	99 (1.8)	221 (1.9)	313 (1.7)	290 (1.3)
Mild sedation, n (%)	3,191 (92)	5,125 (91)	10,455 (91)	16,113 (88)	19,618 (88)
Deep sedation, n (%)	218 (6.3)	388 (6.9)	808 (7.0)	1,809 (9.9)	2,447 (11)
Other, n (%)	0	0	0	23 (<1)	75 (<1)
**Familial risk**					
**Total colonoscopies, n**	**709**	**1,022**	**2,147**	**4,156**	**5,329**
**Unique endoscopy units, n**	**6**	**8**	**20**	**26**	**35**
No medication, n (%)	7 (1.0)	18 (1.8)	39 (1.8)	61 (1.5)	60 (1.1)
Mild sedation, n (%)	653 (92)	909 (89)	1,925 (90)	3,633 (87)	4,662 (88)
Deep sedation, n (%)	49 (6.9)	95 (9.3)	183 (8.5)	460 (11)	586 (11)
Other, n (%)	0	0	0	2 (<1)	21 (<1)
**IBD**					
**Total colonoscopies, n**	**710**	**928**	**1,519**	**2,649**	**3,339**
**Unique endoscopy units, n**	**7**	**7**	**15**	**22**	**29**
No medication, n (%)	7 (1.0)	10 (1.1)	15 (1.0)	27 (1.0)	39 (1.2)
Mild sedation, n (%)	621 (88)	781 (84)	1,201 (79)	2,023 (76)	2,489 (75)
Deep sedation, n (%)	82 (12)	137 (15)	303 (20)	584 (22)	763 (23)
Other, n (%)	0	0	0	15 (<1)	48 (<1)

**Surveillance IBD**					
**Total colonoscopies, n**	**451**	**709**	**1,622**	**2,475**	**2,966**
**Unique endoscopy units, n**	**7**	**8**	**17**	**27**	**34**
No medication, n (%)	5 (1.1)	7 (1.0)	59 (3.6)	54 (2.2)	42 (1.4)
Mild sedation, n (%)	397 (88)	609 (86)	1,332 (82)	2,034 (82)	2,375 (80)
Deep sedation, n (%)	49 (11)	93 (13)	231 (14)	382 (15)	529 (18)
Other, n (%)	0	0	0	5 (0.2)	20 (0.7)
**Symptoms and other**					
**Total colonoscopies, n**	**7,146**	**11,138**	**19,003**	**35,181**	**42,398**
**Unique endoscopy units, n**	**7**	**8**	**21**	**28**	**36**
No medication, n (%)	98 (1.4)	194 (1.7)	378 (2.0)	473 (1.3)	396 (0.9)
Mild sedation, n (%)	6,730 (94)	10,390 (93)	17,487 (92)	32,250 (92)	38,756 (91)
Deep sedation, n (%)	318 (4.5)	554 (5.0)	1,138 (6.0)	2,427 (6.9)	3,128 (7.4)
Other, n (%)	0	0	0	31 (0.1)	118 (0.3)
**Therapeutic**					
**Total colonoscopies, n**	**489**	**632**	**1,120**	**1,630**	**1,712**
**Unique endoscopy units, n**	**7**	**8**	**20**	**27**	**35**
No medication, n (%)	31 (6.3)	21 (3.3)	113 (10)	132 (8.1)	104 (6.1)
Mild sedation, n (%)	320 (65)	366 (58)	553 (49)	683 (42)	710 (42)
Deep sedation, n (%)	138 (28)	245 (39)	454 (41)	799 (49)	873 (51)
Other, n (%)	0	0	0	16 (1.0)	25 (1.5)
**All indications**					
**Total colonoscopies, n**	**15,168**	**23,301**	**44,584**	**81,686**	**95,188**
**Unique endoscopy units, n**	**10**	**9**	**21**	**29**	**36**
No medication, n (%)	240 (1.6)	383 (1.6)	920 (2.1)	1,199 (1.5)	1,022 (1.1)
Mild sedation, n (%)	14,048 (93)	21,367 (92)	40,371 (91)	73,316 (90)	84,378 (89)
Deep sedation, n (%)	880 (5.8)	1,551 (6.7)	3,293 (7.4)	7,078 (8.7)	9,478 (10)
Other, n (%)	0	0	0	93 (<1)	310 (<1)

FIT, fecal immunochemical test; CRC, colorectal cancer; IBD, inflammatory bowel disease.

Table S3. Percentual distribution of the type of applied sedation for individual endoscopy units across different categorized colonoscopy indications

				Sedation type, %†			
Endoscopy unit	Indication category	Included years*	Colonoscopies, n	None	M-O	Prop-ofol	Other
EU2	FIT positive	2023	112	0	98.2	1.8	0
EU3	FIT positive	2023-2024	580	0.7	97.9	1.4	0
EU5	FIT positive	2022-2024	10,565	0.5	99.5	0	0
EU10	FIT positive	2020-2023	1,413	0.4	99.2	0.4	0
EU11	FIT positive	2024	403	0.2	72.5	27.3	0
EU14	FIT positive	2024	917	0	99.9	0.1	0
EU16	FIT positive	2023-2024	575	0.5	99.5	0	0
EU18	FIT positive	2024	447	2.2	95.7	2.0	0
EU19	FIT positive	2023-2024	1,16	0.2	90.9	9.0	0
EU21	FIT positive	2020-2024	2,693	2.8	94.1	3.1	0
EU23	FIT positive	2020-2023	1,585	0.8	98.7	0.5	0
EU28	FIT positive	2022-2024	1,166	0.1	87.3	12.6	0.0
EU30	FIT positive	2023-2024	267	10.5	88.0	0	1.5
EU31	FIT positive	2022-2024	612	0.2	98.2	1.6	0
EU32	FIT positive	2024	204	0.5	92.2	7.4	0
EU33	FIT positive	2024	757	0	98.5	1.5	0
EU34	FIT positive	2023-2024	1,165	0.1	62.0	37.9	0
EU36	FIT positive	2022-2024	2,062	0.2	98.2	1.6	0
EU39	FIT positive	2020-2024	2,453	0.6	98.5	1.0	0
EU40	FIT positive	2022-2024	2,834	2.9	97.1	0	0
EU42	FIT positive	2020-2023	2,189	1.0	97.8	1.2	0
EU43	FIT positive	2023-2024	1,207	0	90.3	9.7	0
EU45	FIT positive	2024	397	1.5	1.0	97.5	0
EU46	FIT positive	2022-2024	2,184	0.4	87.0	12.6	0
EU48	FIT positive	2022-2024	1,129	0	92.6	7.4	0
EU49	FIT positive	2022-2024	3,466	0	98.5	1.5	0
EU50	FIT positive	2024	401	0.2	99.3	0.5	0
EU52	FIT positive	2021-2023	889	1.1	97.5	1.3	0
EU56	FIT positive	2024	96	0	99.0	1.0	0
EU57	FIT positive	2020-2023	2,225	1.4	97.5	1.1	0
EU58	FIT positive	2022-2023	707	0.4	98.9	0.7	0
EU60	FIT positive	2022-2024	563	2.5	97.5	0	0
EU2	Surveillance polyps or CRC	2023	404	0.5	97.5	2.0	0
EU3	Surveillance polyps or CRC	2022-2024	720	1.4	91.8	6.8	0
EU5	Surveillance polyps or CRC	2022-2024	4,788	0.5	99.5	0	0
EU10	Surveillance polyps or CRC	2020-2024	2,224	0.5	93.7	5.8	0
EU14	Surveillance polyps or CRC	2024	941	0	96.4	3.6	0
EU15	Surveillance polyps or CRC	2022-2024	1,433	0.1	84.6	15.4	0
EU16	Surveillance polyps or CRC	2024	218	0.5	99.5	0	0
EU18	Surveillance polyps or CRC	2024	559	6.1	90.3	3.6	0
EU19	Surveillance polyps or CRC	2023-2024	652	0	90.3	9.7	0
EU21	Surveillance polyps or CRC	2020-2024	6,747	1.5	90.8	7.3	0.4
EU23	Surveillance polyps or CRC	2020-2024	2,511	1.2	94.1	4.6	0
EU27	Surveillance polyps or CRC	2024	57	0	80.7	19.3	0
EU28	Surveillance polyps or CRC	2022-2024	3,216	0.1	81.0	18.9	0
EU30	Surveillance polyps or CRC	2023-2024	1,355	17.9	76.8	1.0	4.4
EU31	Surveillance polyps or CRC	2022-2024	638	0.8	94.2	5.0	0
EU32	Surveillance polyps or CRC	2024	153	0	81.0	19.0	0
EU33	Surveillance polyps or CRC	2024	692	0	90.6	9.4	0
EU34	Surveillance polyps or CRC	2023-2024	1,722	0.2	61.4	38.4	0
EU36	Surveillance polyps or CRC	2022-2024	1,978	0.1	88.9	11.0	0
EU39	Surveillance polyps or CRC	2020-2024	3,235	0.9	90.9	8.2	0

EU40	Surveillance polyps or CRC	2021-2024	3,401	8.2	91.8	0	0
EU42	Surveillance polyps or CRC	2020-2024	4,811	0.5	93.8	5.8	0
EU43	Surveillance polyps or CRC	2023-2024	1,978	0.1	85.2	14.7	0
EU45	Surveillance polyps or CRC	2024	330	0	0.3	99.4	0.3
EU46	Surveillance polyps or CRC	2022-2024	2,585	0.7	66.0	33.3	0
EU48	Surveillance polyps or CRC	2022-2024	1,148	0.2	85.9	13.9	0
EU49	Surveillance polyps or CRC	2022-2024	3,120	0	97.5	2.4	0
EU50	Surveillance polyps or CRC	2024	885	0.2	97.7	2.0	0
EU51	Surveillance polyps or CRC	2024	134	1.5	90.3	8.2	0
EU52	Surveillance polyps or CRC	2021-2024	1,414	0.8	92.7	6.4	0
EU54	Surveillance polyps or CRC	2024	186	2.2	87.6	10.2	0
EU56	Surveillance polyps or CRC	2024	183	1.1	94.5	4.4	0
EU57	Surveillance polyps or CRC	2020-2024	5,227	2.0	89.3	8.5	0.2
EU58	Surveillance polyps or CRC	2022-2023	954	0.2	99.2	0.6	0
EU60	Surveillance polyps or CRC	2022-2024	490	4.1	88.8	7.1	0
EU2	Familial CRC risk	2023	72	0	97.2	2.8	0
EU5	Familial CRC risk	2023-2024	1,513	0.8	99.2	0	0
EU10	Familial CRC risk	2022-2024	574	0.7	91.1	8.2	0
EU14	Familial CRC risk	2024	207	0	94.2	5.8	0
EU15	Familial CRC risk	2023-2024	212	0	81.1	18.9	0
EU18	Familial CRC risk	2024	87	4.6	89.7	5.7	0
EU19	Familial CRC risk	2024	115	0	85.2	14.8	0
EU21	Familial CRC risk	2020-2024	1,217	1.2	91.2	7.3	0.3
EU23	Familial CRC risk	2020-2024	439	3.4	89.1	7.5	0
EU28	Familial CRC risk	2022-2024	486	0	75.9	24.1	0
EU30	Familial CRC risk	2023-2024	279	14.0	80.6	1.4	3.9
EU31	Familial CRC risk	2022-2024	133	0	88.0	12.0	0
EU33	Familial CRC risk	2024	131	0	93.9	6.1	0
EU34	Familial CRC risk	2023-2024	350	0	68.6	31.4	0
EU36	Familial CRC risk	2023-2024	412	0.2	81.8	18.0	0
EU39	Familial CRC risk	2020-2024	713	1.0	86.7	12.3	0
EU40	Familial CRC risk	2022-2024	474	9.1	90.9	0	0
EU42	Familial CRC risk	2020-2024	945	0	94.2	5.8	0
EU43	Familial CRC risk	2023-2024	256	0	82.4	17.6	0
EU46	Familial CRC risk	2022-2024	706	0.7	63.5	35.8	0
EU48	Familial CRC risk	2022-2024	176	0	77.8	22.2	0
EU49	Familial CRC risk	2022-2024	630	0	96.7	3.3	0
EU50	Familial CRC risk	2024	118	0	97.5	2.5	0
EU51	Familial CRC risk	2024	113	2.7	77.0	20.4	0
EU52	Familial CRC risk	2021-2024	273	1.1	89.4	9.5	0
EU54	Familial CRC risk	2024	129	1.6	79.8	18.6	0
EU57	Familial CRC risk	2020-2024	1,422	1.1	92.0	6.4	0.6
EU58	Familial CRC risk	2022-2023	215	1.4	96.3	2.3	0
EU60	Familial CRC risk	2022-2024	439	2.3	86.1	11.6	0
EU2	IBD	2023	63	1.6	96.8	1.6	0
EU10	IBD	2022-2024	268	0	90.7	9.3	0
EU14	IBD	2024	199	0	93.5	6.5	0
EU18	IBD	2024	70	8.6	85.7	5.7	0
EU19	IBD	2023-2024	248	0	85.5	14.5	0
EU21	IBD	2020-2024	1,586	1.1	75.5	22.1	1.3
EU23	IBD	2020-2024	953	0.4	88.9	10.7	0
EU28	IBD	2022-2024	488	0	63.5	36.5	0
EU30	IBD	2023-2024	280	12.1	73.9	0.7	13.2
EU32	IBD	2024	58	0	51.7	48.3	0
EU33	IBD	2024	78	0	88.5	11.5	0
EU34	IBD	2023-2024	325	0.3	67.1	32.6	0
EU36	IBD	2023-2024	238	0.8	66.4	32.8	0
EU39	IBD	2020-2024	784	1.0	87.5	11.5	0
EU42	IBD	2023-2024	124	0	91.9	8.1	0
EU43	IBD	2023-2024	245	0.4	85.7	13.9	0

EU46	IBD	2022-2024	741	0.7	59.6	39.7	0
EU52	IBD	2024	70	0	90	10	0
EU57	IBD	2020-2024	886	0.8	83.2	15.5	0.6
EU60	IBD	2022-2024	779	1.2	63.7	35.2	0
EU10	Surveillance IBD	2022-2024	207	0	87.0	13.0	0
EU14	Surveillance IBD	2024	71	0	93.0	7.0	0
EU18	Surveillance IBD	2024	62	9.7	77.4	12.9	0
EU21	Surveillance IBD	2020-2024	942	1.1	84.1	14.0	0.8
EU23	Surveillance IBD	2020-2023	311	1.6	88.7	9.6	0
EU28	Surveillance IBD	2022-2024	491	0	63.5	36.5	0
EU30	Surveillance IBD	2023-2024	176	16.5	75.0	0.6	8.0
EU33	Surveillance IBD	2024	66	0	93.9	6.1	0
EU34	Surveillance IBD	2023-2024	146	0	59.6	40.4	0
EU36	Surveillance IBD	2023-2024	369	0	82.1	17.9	0
EU39	Surveillance IBD	2020-2024	337	0.3	84.3	15.4	0
EU40	Surveillance IBD	2022-2023	293	25.9	74.1	0	0
EU42	Surveillance IBD	2021-2024	279	0.4	92.1	7.5	0
EU43	Surveillance IBD	2023-2024	377	0	83.3	16.7	0
EU46	Surveillance IBD	2022-2024	313	1.6	57.5	40.9	0
EU49	Surveillance IBD	2022-2024	795	0	97.9	2.1	0
EU51	Surveillance IBD	2024	131	3.8	73.3	22.9	0
EU52	Surveillance IBD	2022-2024	193	0.5	92.2	7.3	0
EU54	Surveillance IBD	2024	89	2.2	79.8	18.0	0
EU57	Surveillance IBD	2020-2024	1,224	0.8	87.3	11.7	0.2
EU60	Surveillance IBD	2022-2024	572	1.0	73.1	25.9	0
EU2	Symptoms and other	2023	521	0.2	98.7	1.0	0.2
EU3	Symptoms and other	2022-2024	625	0	96.0	4.0	0
EU5	Symptoms and other	2022-2024	18,642	0.3	99.7	0	0
EU10	Symptoms and other	2020-2024	4,075	0.6	96.1	3.3	0
EU14	Symptoms and other	2024	2,095	0	98.2	1.8	0
EU15	Symptoms and other	2022-2024	2,396	0.1	86.8	13.1	0
EU16	Symptoms and other	2023-2024	1,235	0.2	99.8	0	0
EU18	Symptoms and other	2024	1,489	4.0	93.4	2.6	0
EU19	Symptoms and other	2023-2024	1,701	0	89.2	10.8	0
EU21	Symptoms and other	2020-2024	1,259	1.1	90.9	7.4	0.5
EU23	Symptoms and other	2020-2024	6,567	1.1	94.2	4.7	0
EU27	Symptoms and other	2024	131	0.8	87.0	12.2	0
EU28	Symptoms and other	2022-2024	3,736	0.4	81.5	18.1	0
EU30	Symptoms and other	2023-2024	2,068	12.3	84.1	0.2	3.3
EU31	Symptoms and other	2022-2024	1,076	0.3	93.9	5.9	0
EU32	Symptoms and other	2024	413	0.7	82.1	17.2	0
EU33	Symptoms and other	2024	887	0.1	96.1	3.8	0
EU34	Symptoms and other	2023-2024	2,687	0.3	61.0	38.7	0
EU36	Symptoms and other	2022-2024	3,015	0.6	93.2	6.2	0
EU39	Symptoms and other	2020-2024	5,622	0.9	93.9	5.2	0
EU40	Symptoms and other	2021-2024	5,354	7.3	92.7	0	0
EU42	Symptoms and other	2020-2024	5,146	0.6	96.5	2.9	0
EU43	Symptoms and other	2023-2024	2,069	0	81.8	18.1	0
EU45	Symptoms and other	2024	402	1.2	12.7	86.1	0
EU46	Symptoms and other	2022-2024	5,578	1.1	76.2	22.7	0
EU48	Symptoms and other	2022-2024	1,923	0	87.5	12.5	0
EU49	Symptoms and other	2022-2024	4,856	0	98.3	1.7	0
EU50	Symptoms and other	2024	925	0.5	97.3	2.2	0
EU51	Symptoms and other	2024	223	1.8	87.0	11.2	0
EU52	Symptoms and other	2021-2024	3,827	2.0	94.7	3.3	0
EU53	Symptoms and other	2024	200	0	100	0	0
EU54	Symptoms and other	2024	149	0.7	91.3	8.1	0
EU55	Symptoms and other	2024	186	0	96.2	3.8	0
EU56	Symptoms and other	2024	367	0.3	97.3	2.5	0
EU57	Symptoms and other	2020-2024	9,778	2.0	93.8	4.1	0.1

EU58	Symptoms and other	2022-2023	868	0.8	98.4	0.8	0
EU60	Symptoms and other	2022-2024	1,321	3.3	87.3	9.5	0
EU15	Therapeutic	2023	61	0	41.0	59.0	0
EU21	Therapeutic	2020-2024	1,388	5.4	60.2	34.1	0.2
EU28	Therapeutic	2022-2024	245	0.4	34.7	64.9	0
EU30	Therapeutic	2023-2024	171	28.1	50.3	3.5	18.1
EU36	Therapeutic	2023-2024	184	0.5	10.3	89.1	0
EU39	Therapeutic	2020-2022	231	2.2	76.2	21.6	0
EU40	Therapeutic	2022-2023	256	68.8	30.9	0.4	0
EU42	Therapeutic	2021-2024	322	0	28.9	71.1	0
EU43	Therapeutic	2023-2024	246	0.4	48.8	50.8	0
EU46	Therapeutic	2023-2024	160	0.6	19.4	80	0
EU49	Therapeutic	2022-2024	226	0	83.2	16.8	0
EU51	Therapeutic	2024	52	11.5	30.8	57.7	0
EU54	Therapeutic	2024	51	0	7.8	92.2	0
EU57	Therapeutic	2021-2024	483	2.3	40.8	55.5	1.4
EU60	Therapeutic	2022-2024	410	0.7	42.9	56.3	0
EU2	All indications	2020-2023	1,268	0.3	96.8	2.8	0.1
EU3	All indications	2022-2024	2,088	0.8	93.6	5.6%	0.0%
EU4	All indications	2020-2021	58	1.7	88.7		
EU5	All indications	2022-2024	35,693	0.4	99.6	0	0.0%
EU8	All indications	2023	25	NA	NA	NA	NA
EU10	All indications	2020-2024	9,058	0.5	94.2	5.3	0.0%
EU11	All indications	2024	405	0.2	72.6	27.2	0.0%
EU13	All indications	2023	40	NA	NA	NA	NA
EU14	All indications	2024	4,476	0.0	97.3	2.7	0.0%
EU15	All indications	2022-2024	4,182	0.1	84.6	15.4	0.0%
EU16	All indications	2023-2024	2,154	0.3	99.7	0	0.0%
EU18	All indications	2024	2,761	4.8	91.9	3.3	0.0%
EU19	All indications	2023-2024	4,003	0.0	89.2	10.8	0.0%
EU21	All indications	2020-2024	27,163	1.6	88.5	9.4	0.5%
EU23	All indications	2020-2024	12,524	1.2	93.8	5.0	0.0%
EU27	All indications	2024	229	0.4	82.5	17.0	0.0%
EU28	All indications	2022-2024	9,828	0.2	78.8	21.0	0.0%
EU30	All indications	2022-2024	4,596	14.7	79.7	0.7	4.9%
EU31	All indications	2022-2024	2,599	0.4	93.6	6.0	0.0%
EU32	All indications	2024	923	0.5	80.1	19.4	0.0%
EU33	All indications	2023-2024	2,639	0.0	94.6	5.3	0.0%
EU34	All indications	2023-2024	6,488	0.2	61.3	38.5	0.0%
EU35	All indications	2022	39	NA	NA	NA	NA
EU36	All indications	2022-2024	8,331	0.3	89.6	10.1	0.0%
EU39	All indications	2020-2024	13,409	0.9	92.7	6.5	0.0%
EU40	All indications	2020-2024	12,719	8.5	91.5	0	0.0%
EU42	All indications	2020-2024	14,025	0.6	93.7	5.7	0.0%
EU43	All indications	2023-2024	6,378	0.1	83.5	16.4	0.0%
EU45	All indications	2024	1,240	1.0	5.1	93.8	0.1%
EU46	All indications	2022-2024	12,311	0.8	72.8	26.3	0.0%
EU48	All indications	2022-2024	4,625	0	86.8	13.1	0.0%
EU49	All indications	2022-2024	13,124	0	97.8	2.2	0.0%
EU50	All indications	2024	2,370	0.3	97.5	2.2	0.0%
EU51	All indications	2024	653	3.1	78.7	18.2	0.0%
EU52	All indications	2021-2024	6,927	1.5	93.9	4.6	0.0%
EU53	All indications	2024	229	0	100	0	0.0%
EU54	All indications	2023-2024	713	1.5	79.2	19.2	0.0%
EU55	All indications	2024	290	0.0	91.0	9.0	0.0%
EU56	All indications	2024	698	0.4	96.1	3.4	0.0%
EU57	All indications	2020-2024	21,259	1.8	90.9	7.1	0.2%
EU58	All indications	2022-2023	2,813	0.5	98.5	1.0	0.0%
EU60	All indications	2022-2024	4,574	2.3	78.8	18.9	0.0%

M-O, midazolam- and/or opiod-based sedation; EU, endoscopy unit; FIT, fecal immunochemical test; CRC, colorectal cancer; IBD, inflammatory bowel disease.

*Data are shown for units with at least 50 procedures recorded within a given indication category per calendar year only;
†In case of available data for multiple years, the reported percentages reflect means as calculated based on available data for each of the different years.

Table S4. Mean dose of administered midazolam across different categorized colonoscopy indications, reported at the level of individual endoscopy units

	FIT positive		Surveillance polyps or CRC		Familial risk		IBD		Surveillance IBD		Symptoms and other		Therapeutic	
Endoscopy unit	n	mean (±sd)*	n	mean (±sd)*	n	mean (±sd)*	n	mean (±sd)*	n	mean (±sd)*	n	mean (±sd)*	n	mean (±sd)*
**EU2**	NA	NA	NA	NA	NA	NA	2	NA	NA	NA	2	NA	1	NA
**EU3**	562	3.6 (1.1)	655	2.9 (0.9)	94	NA	NA	NA	9	NA	592	3.4 (1.2)	21	NA
**EU4**	8	NA	16	NA	NA	NA	2	NA	2	NA	22	NA	1	NA
**EU5**	9,880	3.6 (1.1)	3,665	3.5 (1.2)	1,128	3.9 (1.2)	57	NA	20	NA	14,217	3.8 (1.3)	28	NA
**EU6**	1,909	4.0 (1.1)	NA	NA	NA	NA	NA	NA	NA	NA	NA	NA	NA	NA
**EU7**	32	NA	788	2.6 (0.6)	595	3.2 (1.2)	18	NA	197	3.3 (1.2)	625	2.9 (1.0)	24	NA
**EU8**	1	NA	4	NA	NA	NA	NA	NA	4	NA	3	NA	NA	NA
**EU9**	503	3.2 (0.9)	1,164	3.2 (0.9)	392	3.6 (1.1)	NA	NA	NA	NA	575	3.4 (1.1)	155	3.6 (1.1)
**EU10**	1,402	4.4 (0.8)	2,082	3.6 (0.9)	585	3.8 (0.9)	279	4.3 (1.1)	239	4.6 (0.8)	3,914	3.8 (1.0)	25	NA
**EU11**	975	3.2 (0.9)	80	3.2 (1.1)	12	NA	NA	NA	5	NA	23	NA	NA	NA
**EU12**	681	4.4 (1.1)	351	4.6 (0.8)	41	NA	181	4.9 (1.1)	26	NA	621	4.7 (0.9)	27	NA
**EU13**	NA	NA	5	NA	NA	NA	NA	NA	2	NA	28	NA	NA	NA
**EU14**	912	3.0 (0.8)	903	2.6 (0.7)	193	3.0 (1.0)	186	3.7 (1.2)	66	NA	2,045	2.8 (1.1)	26	NA
**EU15**	NA	NA	1,204	2.5 (0.3)	187	2.6 (0.4)	NA	NA	20	NA	2,061	2.5 (0.5)	38	NA
**EU16**	1,354	3.0 (0.9)	294	2.9 (0.9)	82	NA	27	NA	NA	NA	1,921	3.2 (1.1)	18	NA
**EU17**	1,322	3.7 (1.2)	970	3.5 (1.2)	185	4.2 (1.2)	146	4.4 (1.1)	163	4.5 (1.4)	2,806	3.7 (1.3)	58	NA
**EU18**	897	3.4 (0.9)	1,988	3.4 (1.0)	312	4.1 (1.2)	99	NA	151	4.0 (1.1)	4,327	3.6 (1.2)	68	NA
**EU19**	1,769	5.4 (1.4)	584	5.2 (1.2)	139	5.9 (1.8)	210	7.0 (2.4)	38	NA	1,503	5.8 (1.9)	13	NA
**EU20**	1,018	4.6 (0.8)	602	4.5 (0.9)	96	NA	NA	NA	41	NA	1,071	4.5 (1.0)	36	NA
**EU21**	2,477	2.8 (0.7)	6,001	2.5 (0.7)	1,095	2.9 (0.9)	1,159	3.3 (1.2)	778	3.2 (1.2)	11,087	2.7 (0.9)	788	2.7 (0.7)
**EU22**	1	NA	329	2.9 (0.9)	68	NA	NA	NA	41	NA	860	3.0 (1.1)	25	NA
**EU23**	1,560	3.7 (0.8)	2,349	3.7 (0.9)	388	4.3 (0.9)	845	4.3 (1.0)	313	4.2 (0.9)	6,168	3.7 (1.0)	74	NA
**EU24**	593	4.7 (0.8)	NA	NA	NA	NA	NA	NA	NA	NA	NA	NA	NA	NA
**EU25**	106	4.7 (0.7)	NA	NA	NA	NA	NA	NA	NA	NA	4	NA	NA	NA
**EU28**	462	2.5 (1.3)	695	2.3 (1.1)	93	NA	104	2.4 (0.4)	84	NA	1,063	2.5 (1.5)	31	NA
**EU29**	601	3.3 (1.2)	89	NA	17	NA	21	NA	12	NA	290	3.8 (1.5)	7	NA
**EU30**	186	2.4 (0.7)	622	1.8 (0.9)	80	NA	136	3.2 (1.2)	73	NA	947	2.0 (1.2)	57	NA
**EU31**	621	3.9 (1.2)	592	2.9 (1.1)	136	3.7 (1.3)	35	NA	31	NA	1,012	3.2 (1.4)	17	NA

**EU32**	185	2.5 (0.1)	123	2.8 (0.8)	29	NA	30	NA	25	NA	336	2.9 (0.8)	4	NA
**EU33**	1,188	4.3 (1.0)	926	3.9 (1.1)	192	4.4 (1.1)	69	NA	87	NA	1,263	4.3 (1.5)	5	NA
**EU34**	999	4.8 (1.0)	1,055	4.4 (1.3)	238	5.1 (1.1)	216	5.6 (1.4)	86	NA	1,629	4.7 (1.5)	11	NA
**EU35**	NA	NA	17	NA	NA	NA	NA	NA	NA	NA	20	NA	1	NA
**EU36**	2,042	3.8 (1.1)	1,755	3.8 (1.2)	346	4.3 (1.4)	159	5.1 (1.5)	327	4.8 (1.6)	2,609	4.1 (1.5)	23	NA
**EU37**	251	4.3 (1.6)	509	4.0 (0.9)	56	NA	NA	NA	50	4 NA	908	4.2 (1.0)	3	NA
**EU38**	1,224	4.4 (1.1)	NA	NA	NA	NA	NA	NA	NA	NA	NA	NA	NA	NA
**EU39**	1,026	4.1 (1.2)	1,491	3.5 (1.4)	278	4.4 (1.6)	456	4.4 (1.4)	148	4.3 (1.0)	2,903	3.7 (1.5)	166	3.5 (1.2)
**EU40**	2,694	3.2 (0.8)	3,014	2.8 (0.8)	410	3.5 (1.1)	NA	NA	256	3.6 (1.2)	4,326	3.1 (1.0)	86	NA
**EU41**	2,038	2.7 (0.7)	NA	NA	NA	NA	NA	NA	NA	NA	NA	NA	NA	NA
**EU42**	2,138	5.3 (1.1)	4,505	4.9 (1.2)	892	5.6 (1.3)	231	6.1 (2.0)	294	5.7 (1.4)	4,944	5.1 (1.5)	111	5.4 (1.9)
**EU43**	1,105	4.5 (0.7)	1,534	4.7 (0.7)	196	4.8 (0.7)	205	5.0 (0.8)	303	4.8 (0.6)	1,604	4.6 (0.9)	117	4.6 (0.7)
**EU45**	3	NA	NA	NA	NA	NA	NA	NA	NA	NA	NA	NA	NA	NA
**EU46**	1,863	4.1 (0.8)	1,673	3.8 (0.9)	439	4.4 (0.8)	435	4.7 (0.8)	174	4.5 (0.7)	4,187	4.1 (0.9)	37	NA
**EU47**	324	5.0 (0.3)	267	4.8 (0.6)	40	NA	NA	NA	33	NA	719	4.7 (0.8)	4	NA
**EU48**	1,068	3.0 (1.2)	966	2.7 (1.0)	136	3.1 (1.1)	38	NA	79	NA	1,660	3.0 (1.4)	48	NA
**EU49**	3,451	4.4 (1.1)	3,031	4.3 (1.4)	606	5.2 (1.7)	30	NA	766	6.2 (2.2)	4,645	4.6 (1.8)	181	4.6 (1.9)
**EU51**	NA	NA	425	2.9 (1.0)	367	3.1 (1.2)	10	NA	506	3.2 (1.3)	851	3.0 (1.2)	81	NA
**EU52**	864	4.7 (0.8)	1,304	4.3 (1.3)	242	4.8 (0.7)	192	4.7 (1.0)	216	4.8 (0.8)	3,600	4.4 (1.2)	43	NA
**EU53**	NA	NA	6	NA	10	NA	5	NA	1	NA	193	4.4 (1.3)	NA	NA
**EU54**	82	NA	178	3.3 (1.0)	107	4.1 (1.7)	37	NA	74	NA	152	3.4 (1.3)	5	NA
**EU55**	1,926	3.0 (0.6)	1,905	3.2 (0.8)	385	4.1 (1.0)	246	4.3 (1.1)	471	4.2 (1.0)	4,649	3.5 (1.0)	76	NA
**EU56**	343	3.0 (0.8)	730	3.0 (0.8)	118	3.3 (0.9)	NA	NA	26	NA	1,468	3.1 (1.1)	30	NA
**EU57**	2,168	3.0 (1.0)	4,623	2.5 (0.8)	1,293	2.9 (1.0)	723	3.3 (1.1)	1,054	3.0 (1.1)	9,063	2.8 (1.1)	190	2.9 (0.9)
**EU58**	376	4.1 (0.8)	687	3.7 (0.8)	125	4.1 (0.9)	NA	NA	29	NA	546	3.9 (1.0)	3	NA
**EU59**	4	NA	115	4.1 (1.7)	40	NA	188	5.2 (2.0)	205	5.2 (1.9)	436	4.3 (2.0)	17	NA
**EU60**	545	4.6 (0.7)	433	4.4 (1.5)	377	4.5 (1.0)	496	4.4 (1.1)	413	4.5 (1.0)	1,142	3.9 (1.2)	175	3.8 (1.2)
**EU61**	265	2.8 (1.2)	404	2.6 (1.1)	69	NA	NA	NA	95	NA	1,773	2.9 (1.3)	21	NA

FIT, fecal immunochemical test; CRC, colorectal cancer; IBD, inflammatory bowel disease; EU, endoscopy unit. *Mean dose was not reported if fewer than 100 procedures were recorded.

SUPPLEMENTARY FIGURES

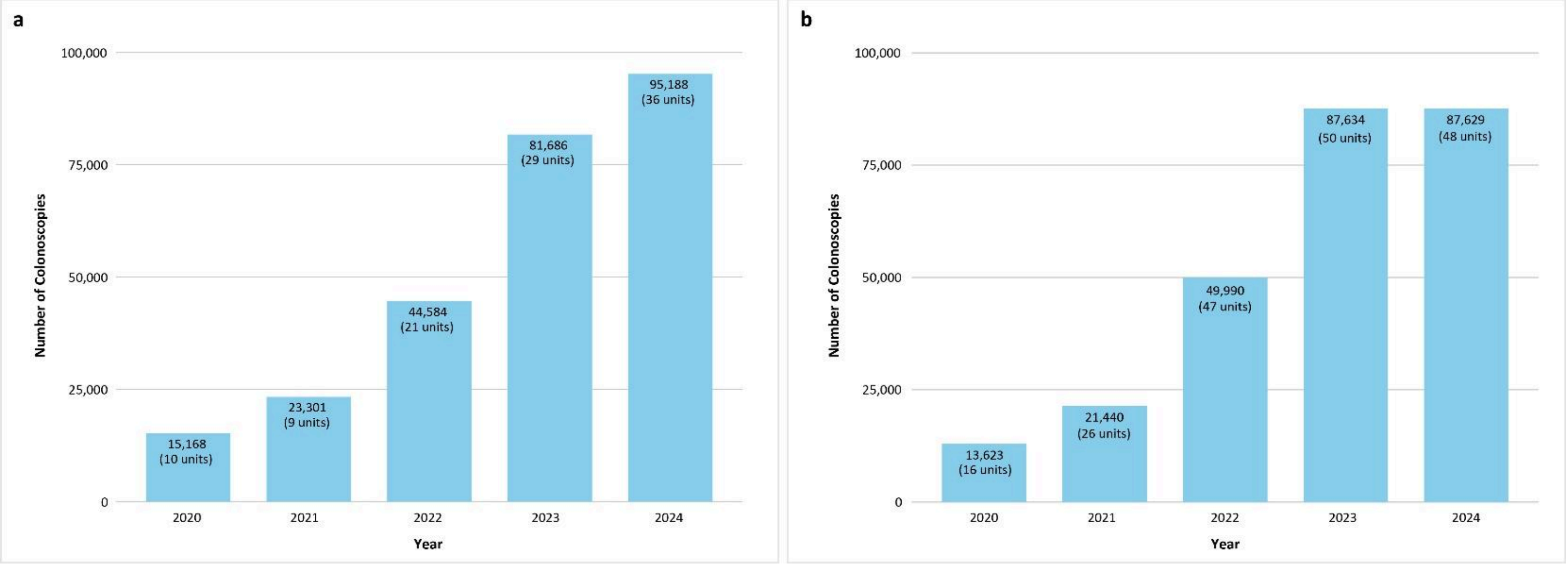

Figure S1. Number of included colonoscopies per year for (a) analyses involving trends and variations in types of applied sedation and (b) analyses involving variations in midazolam dosing regimens

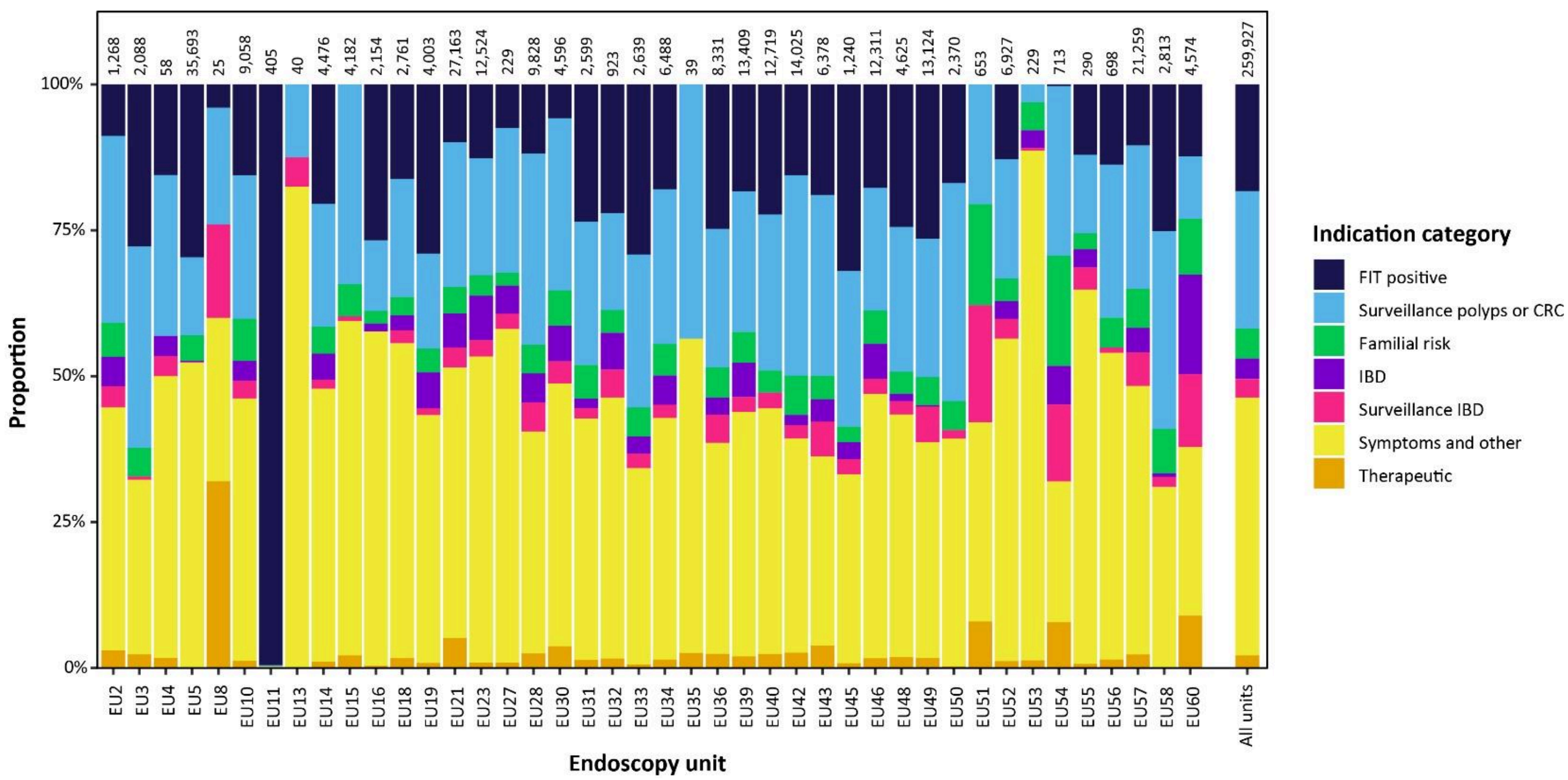

Figure S2. Stacked bar chart illustrating the distribution of colonoscopies by indication category across endoscopy units. The numbers above each bar indicate the total number of procedures included for each of the endoscopy units. Footnotes: EU, endoscopy unit; FIT, fecal immunochemical test; CRC, colorectal cancer; IBD, inflammatory bowel disease.

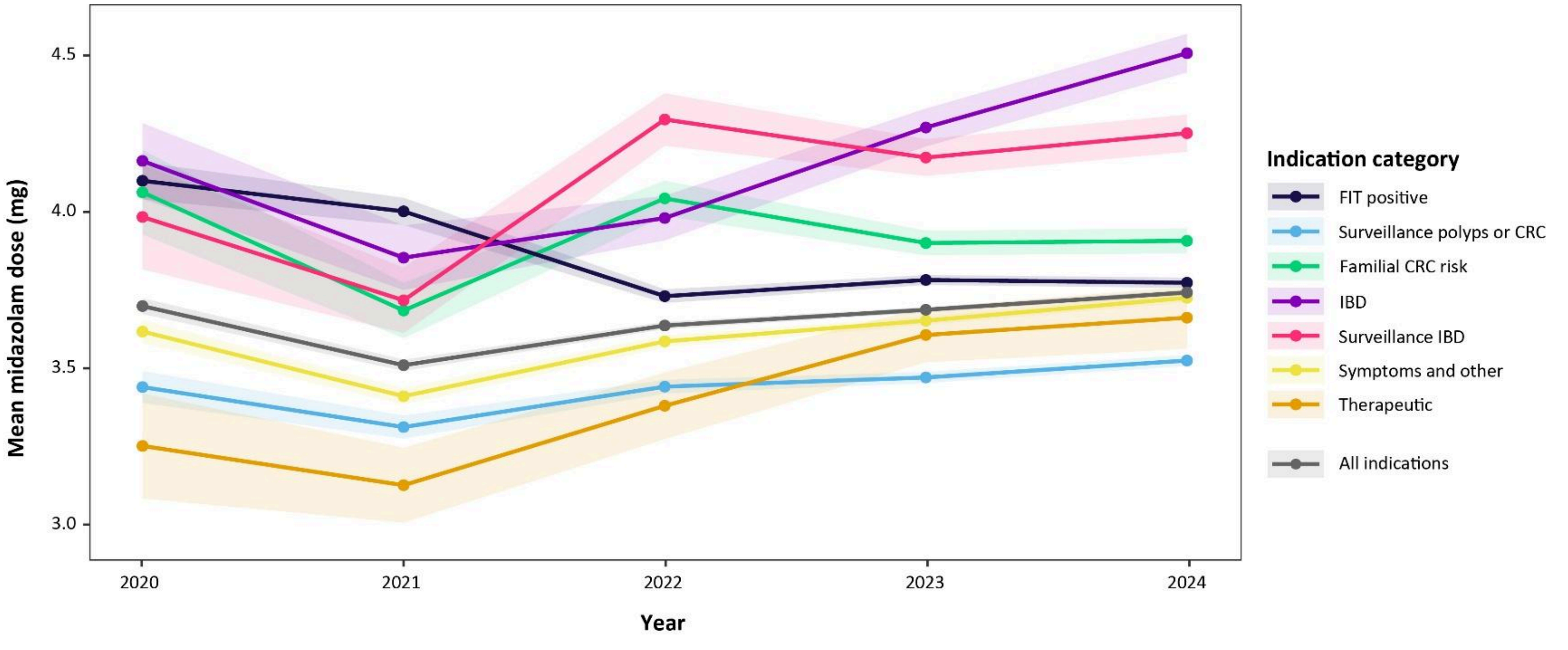

Figure S3. Annual trends (2020 to 2024) in the mean midazolam dose administered for procedures performed under midazolam-based sedation (either without or without co-administration of opioids) across categorized colonoscopy indications. For each calendar year, data points represent the mean midazolam dose calculated from all procedures within a given indication category. Lines connect annual means to illustrate temporal trends. The shaded areas surrounding each line denote the corresponding 95% confidence intervals. Footnotes: FIT, fecal immunochemical test; CRC, colorectal cancer; IBD, inflammatory bowel disease.

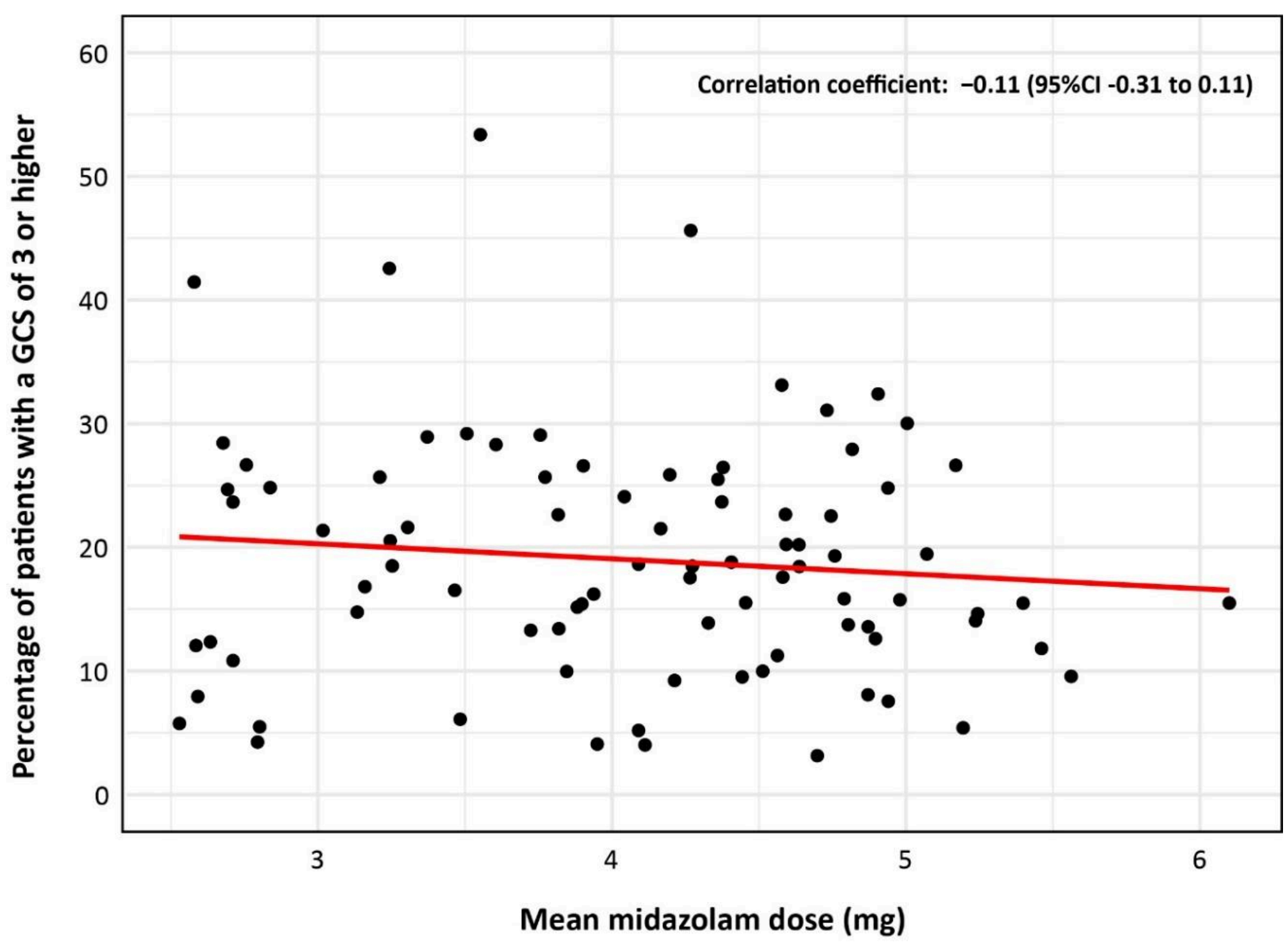

Figure S4. Post-hoc analysis of the correlation between the mean midazolam dose administered for midazolam-based sedation (either with or without co-administration of opioids) and the percentage of patients for whom moderate-to-severe discomfort (Gloucester Comfort Scale score >3) was reported. Included procedures all involved colonoscopies after a positive fecal immunochemical test for the Dutch colorectal cancer screening program [1]. Each point within the plot represents a single endoscopy unit. Units for which fewer than 100 procedures were recorded, were excluded from the analysis. The red line represents the linear regression line corresponding to the Pearson correlation analysis used to calculate the correlation coefficient. Footnotes: GCS, Gloucester Comfort Scale.

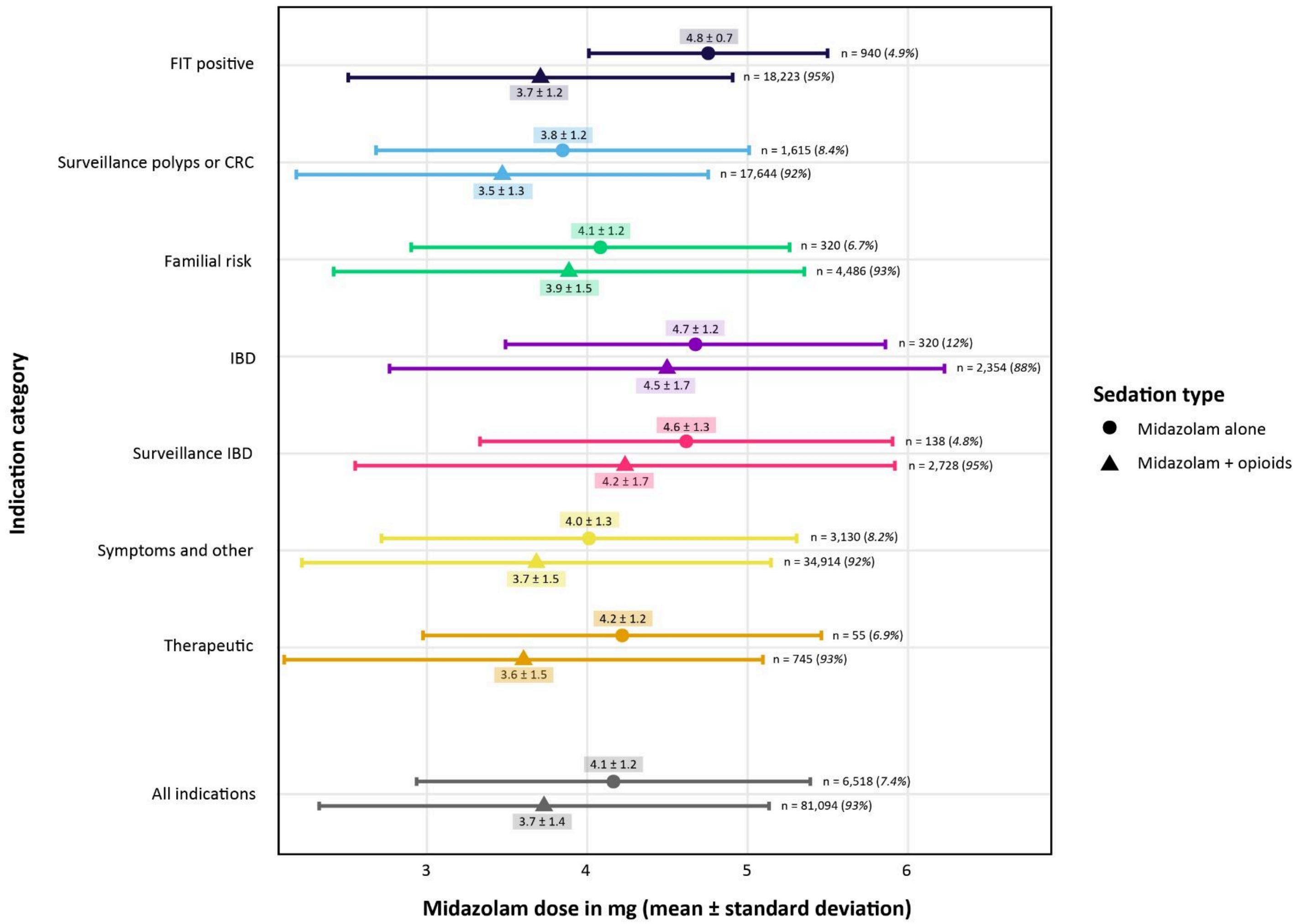

Endosc Int Open | DOI 10.1055/a-2942-3921 | © The Author(s).

Figure S5. Dumbbell plots illustrating the mean (± standard deviation) midazolam dose administered stratified by sedation regimen (midazolam alone vs. midazolam plus opioids) and colonoscopy indication category. The analysis includes all colonoscopies recorded in the DGEA registry in 2024 for which the dose of administered midazolam was specified and during which no agents other than midazolam and/or opioids were administered. Numbers displayed at the right end of each bar represent the total number of included procedures per indication category, with the corresponding percentage indicating the proportion relative to the total number of procedures within the same indication category.

- Footnotes: FIT, fecal immunochemical test; CRC, colorectal cancer; IBD, inflammatory bowel disease.

SUPPLEMENTARY MATERIALS - REFERENCES

1. Nass KJ, van Doorn SC, van der Vlugt M et al. Impact of sedation on the Performance Indicator of Colonic Intubation. Endoscopy 2021; 53: 619–626. doi:10.1055/a-1254-5182
